# Oringano: Shared Ring-Based Gestures for Controlling Internet of Things Devices in a Smart Home

**DOI:** 10.3390/s26175605

**Published:** 2026-09-03

**Authors:** Thanh-Diane Nguyen, Donatien Grolaux, Jean Vanderdonckt

**Affiliations:** 1 ICHEC Research Lab (IRL), Department Management of Information Systems (MIS), ICHEC Brussels Management School, 1150 Woluwé-Saint-Pierre, Belgium or thanhdiane.nguyen@uclouvain.be (T.-D.N.); or donatien.grolaux@uclouvain.be (D.G.); 2 Louvain Research Institute in Management and Organizations (LouRIM), Université catholique de Louvain, 1348 Louvain-la-Neuve, Belgium

**Keywords:** gesture elicitation study, gesture-based user interface, gesture set, human–computer interaction, Internet of Things, microgestures, sensor, smart home, smart rings, 89.20.Ff, 07.07.Df, 68U35, C88

## Abstract

Smart rings are emerging as a promising class of sensing devices that enable unobtrusive, always-available interaction with Internet of Things (IoT) ecosystems. These wearable devices support intuitive gesture-based control while minimizing user attention and preserving mobility. However, despite rapid advances in sensing hardware and gesture recognition algorithms, little is known about how users associate ring-based shared gestures with smart-home commands. To fill this gap, this paper presents Oringano, a framework for designing and evaluating a vocabulary of shared ring-based gestures for controlling IoT devices in a smart home. These gestures are original in that members of the same group can share the same gestures for the same actions, as well as gestures customized by each individual. The proposed approach combines (i) a synthesis of contemporary smart ring-based gesture interaction literature, (ii) a user-centered requirements elicitation identifying representative smart-home control actions, (iii) a gesture elicitation study involving N=30 participants to derive a vocabulary of shared ring-based gestures for 15 IoT control actions, (iv) an empirical analysis of gesture agreement and usability of Oringano, a smartphone prototype for managing shared ring-based gestures, and (v) a set of implications for designing shared gestures for future smart-ring systems. Experimental results demonstrate high agreement for concrete actions such as selection, navigation, and media control, whereas abstract actions exhibit greater variability, highlighting opportunities for personalized gestural interaction. The shared gestures benefit from a higher average agreement rate (+84%), a slightly lower goodness of fit (−13%), and a longer thinking time (+115%) than normal ring-based gestures. The subjective satisfaction resulting from the usability evaluation of Oringano, based on the elicited vocabulary, is overall positive (4.5/5). These results advance the design of next-generation ring-based systems by bridging user-centered gesture interaction with practical sensing technologies for IoT interaction.

## 1. Introduction

Wearable sensing technologies have rapidly evolved over the past decade, enabling continuous [1], unobtrusive [2], and context-aware interaction [3] between users and digital environments. Among wearable devices, smart rings have recently emerged as an attractive interaction platform [4] because of their compact form factor [5], social acceptability [6], low energy consumption [7], and continuous availability during everyday activities [8]. Unlike smartwatches or smartphones [9], smart rings can remain unobtrusive while continuously acquiring motion [10,11], touch [12], magnetic [13], physiological [14], or environmental data [15] through embedded inertial measurement units (IMUs) [16], capacitive sensors, and other miniaturized sensing technologies. These capabilities have stimulated increasing research interest in wearable sensing for health monitoring [14], authentication [17], gesture recognition [9], augmented reality [18], and Internet of Things (IoT) interaction [19,20]. The expansion of IoT devices, as expressed by general public adoption [21] rather than ubiquitous computing, where user interaction may be limited, has simultaneously increased demand for intuitive, ubiquitous interaction [20]. Smart homes integrate connected lighting, climate control, entertainment systems, security devices [22], household appliances, and smart television services [23], requiring users to interact with an increasingly heterogeneous ecosystem that demands consistency across gestures [24]. Vocal interaction is subject to privacy, noisy environments, shared living spaces where complex commands exist, and accessibility restrictions (e.g., Internet reliance) [25]. Similarly, smartphone-based control often requires visual attention and interrupts ongoing activities. Gesture-based interaction [26] offers a promising alternative by allowing users to issue commands while maintaining visual focus on their primary tasks [27].

Recent advances in wearable sensing have significantly improved the accuracy and robustness of gesture recognition [15]. After concentrating on sensing hardware and mastering gesture recognition, comprehensive reviews also reveal considerable fragmentation in interaction techniques and gesture definitions across different smart-ring platforms [4,28]. Little attention has been devoted to understanding how users associate ring-based gestures with control actions. Most existing systems define gesture sets from an engineering perspective, often optimizing recognition performance rather than interaction quality or memorability. Consequently, different devices employ incompatible gesture vocabularies, limiting learnability, interoperability, and standardization. This limitation is particularly important in smart-home environments, where users expect intuitive interaction with numerous heterogeneous IoT devices through a consistent set of commands [29]. Developing user-centered gesture vocabularies therefore remains an open challenge in research [30].

Gesture Elicitation Studies (GESs) [31] have become an established methodology for designing natural interaction vocabularies by involving end users in the gesture creation process rather than prescribing gestures a priori [30] or counting on designer-defined gestures [32]. GESs have been successfully applied across many domains, including touch interfaces, free-hand interaction, virtual and augmented reality, body-centric interfaces, and multimodal systems [33,34]. Agreement-based metrics further enable researchers to quantify consensus among participants and objectively evaluate gesture vocabularies [35]. Only a limited number of elicitation studies have investigated smart-ring interaction.

**Figure 1:**
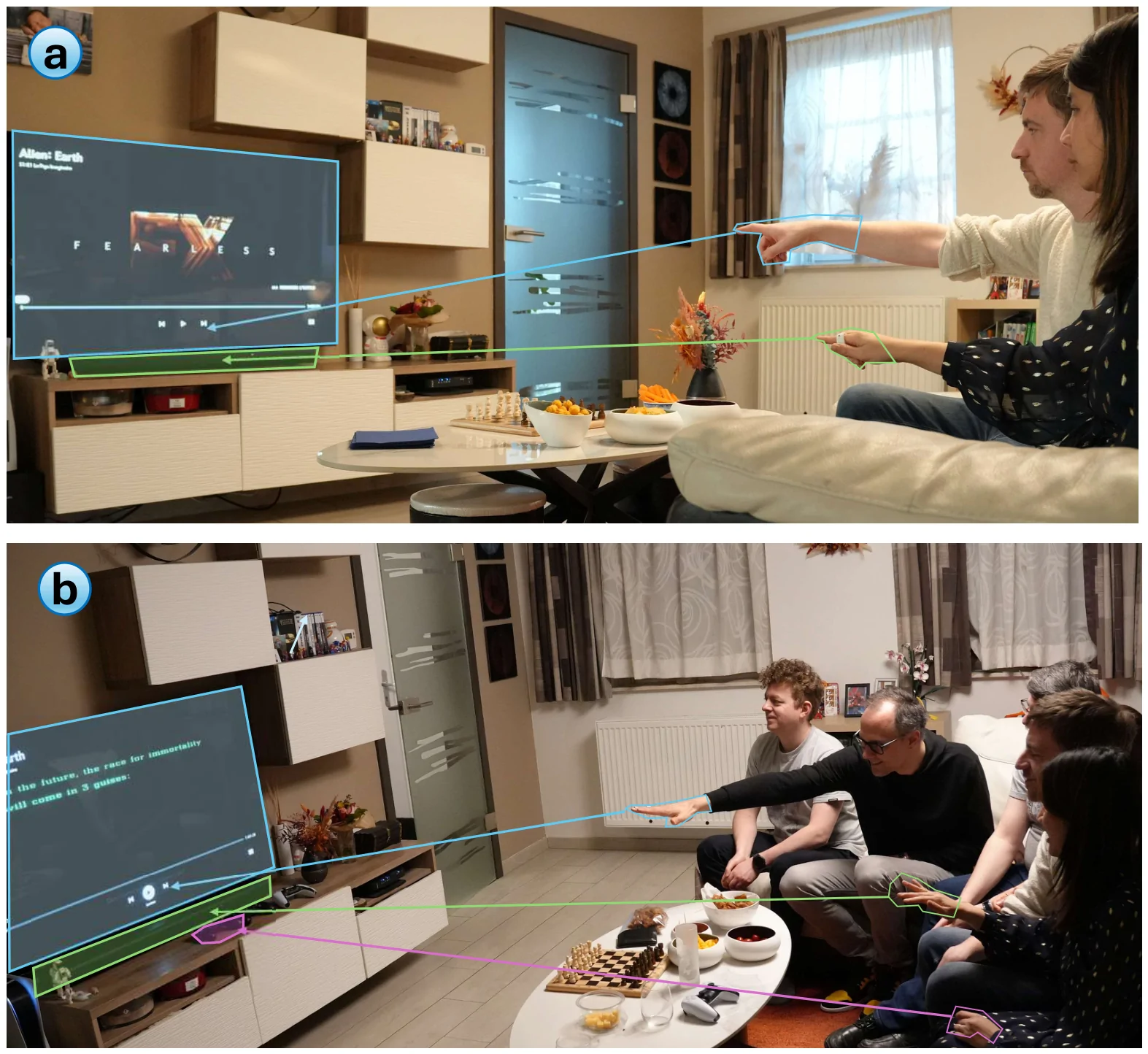
Ring-based gestures shared among users with Oringano: (**a**) A user advances through a movie by pointing the smart ring (in blue) to a smart TV and moving it to the right; a second user increases the volume by pointing the smart ring (in green) to a sound bar and rolling it clockwise. (**b**) A user switches the subwoofer on by pressing the ring to it (in purple), while a second user plays the current movie by selecting the smart ring to play (in blue), and a third user decreases the volume by rolling the smart ring counterclockwise (in green).

To address this research gap, this paper presents Oringano, a framework for designing and evaluating a vocabulary of shared ring-based gestures for controlling IoT devices in a smart home. Rather than proposing another gesture recognition algorithm, Oringano integrates ring-based wearable sensing with gesture elicitation to derive a consensus-based gesture vocabulary grounded in users’ mental models (Figure 1).

The main contributions of this paper are fivefold:

1. A synthesis of the contemporary smart ring-based gesture interaction literature.
2. A user-centered requirements elicitation identifying representative smart-home control actions.
3. A gesture elicitation study involving N=30 participants to derive a vocabulary of shared ring-based gestures for 15 IoT control actions,
4. An empirical analysis of gesture agreement and usability of Oringano, a smartphone prototype for managing shared ring-based gestures.
5. A set of implications for designing shared gestures for future smart-ring systems.

The remainder of this paper is organized as follows. Section 2 reviews related work on wearable sensing, smart rings, gesture elicitation, and IoT interaction, in particular, with a systematic literature review in Section 3. Section 4 presents the requirements elicitation process, while Section 5 reports the gesture elicitation study and consensus analysis. Section 6 describes the prototype implementation and sensing architecture. Section 7 presents the experimental evaluation and its threats to validity. Section 8 suggests five implications for designing shared, ring-based gestures in the context of a smart-home interaction. Section 9 concludes the paper with future research directions.

This paper investigates how users naturally map ring-based gestures to smart-home control actions. Rather than proposing a new recognition algorithm, we focus on understanding the interaction vocabulary that emerges from user expectations. Our work is motivated by four research questions:

*RQ*_1_: What IoT control actions are most relevant for smart-ring interaction in smart-home environments?
*RQ*_2_: What gesture vocabulary do users naturally propose for these actions?
*RQ*_3_: To what extent does the resulting vocabulary demonstrate agreement, memorability, and usability?
*RQ*_4_: How can the elicited vocabulary inform future smart-ring interaction design?

## 2. Related Work

### 2.1. Smart Rings as Wearable Sensing Platforms

Recent advances in sensor miniaturization, low-power electronics, and wireless communication have transformed smart rings into powerful wearable sensing platforms capable of supporting both continuous sensing and interactive applications [28]. Modern smart rings integrate many different types of technologies, including IMUs, accelerometers, gyroscopes, magnetometers, capacitive sensors, temperature sensors, near-field communication (NFC), and Bluetooth Low Energy (BLE), enabling a broad spectrum of applications ranging from health monitoring to ubiquitous Human–Computer Interaction (HCI) [19].

Compared with smartwatches and wrist-worn devices [9], smart rings provide several advantages. Their position on the fingers allows them to capture subtle finger and hand movements [6] with high precision while remaining lightweight, socially acceptable, and suitable for continuous wear. A recent systematic literature review [28] identified more than 200 publications on smart rings and proposed a taxonomy encompassing sensing modalities, interaction techniques, passive sensing, health monitoring, and output mechanisms, demonstrating the rapid growth of this research area while highlighting the fragmentation of interaction techniques across devices [36].

### 2.2. Gesture Recognition with Smart Rings

Gesture recognition constitutes one of the most promising applications of wearable sensing. Instead of relying on external cameras or environmental infrastructures that are constrained by physical conditions (e.g., lighting), smart rings exploit embedded sensors to recognize finger [8,37,38,39], hand [18,40], and wrist [13,41,42] movements directly on the user. Such wearable sensing approaches improve mobility, preserve user privacy, reduce environmental constraints, and maintain reliable performance [8]. Numerous recognition techniques have been investigated, including Dynamic Time Warping (DTW), Hidden Markov Models (HMMs), Support Vector Machines (SVMs), Convolutional Neural Networks (CNNs), and transformers [21,43,44]. Kurz et al. [9] compared gesture recognition using motion sensors embedded in smart rings and smartwatches, demonstrating that the finger location offers superior sensitivity for recognizing fine-grained gestures while maintaining comparable accuracy. Smart rings are suitable as natural input devices.

### 2.3. Gesture Elicitation for Wearable Interaction

Although gesture recognition algorithms continue to improve, defining appropriate gesture vocabularies remains a fundamental challenge [30]. Many systems prescribe gestures designed to maximize recognition accuracy, often overlooking users’ expectations and mental models. Consequently, different devices employ incompatible gesture sets.

Gesture elicitation studies (GES) have become an established user-centered methodology for designing intuitive gesture vocabularies. Introduced by Wobbrock et al. [31], the methodology invites participants to propose gestures for predefined actions, allowing researchers to derive interaction vocabularies directly from users rather than from designers, which is preferable [32]. Agreement-based metrics later proposed by Vatavu and colleagues [35] further quantify the degree of consensus among participants, enabling objective comparisons between elicited gesture sets.

Several gesture elicitation studies have investigated touch interfaces, freehand interaction, virtual reality, augmented reality, public displays, and wearable computing [33,34]. However, relatively few have specifically examined smart rings. Gheran et al. [4,45] conducted one of the first GESs devoted exclusively to smart-ring gestures by analyzing 672 user-defined gestures proposed by 24 participants. This initial GES has been repeated [46] and replicated four times [47]. Their results revealed low agreement rates between users, suggesting that designing universally acceptable ring gestures is more challenging than for other interaction modalities. The low agreement rate and the need for multiple iterations may indicate increased interaction complexity; however, these observations could also be explained by participants’ limited familiarity with smart ring technology. Because smart-ring interaction remains relatively novel, it cannot be determined from the available data alone whether the observed effect reflects the inherent complexity of the modality or users’ limited prior experience with smart ring technology. They also demonstrated that methodological factors—including participant characteristics, grouping criteria, and experimental conditions—significantly influence agreement measures.

More recently, TapStrapGest  [48] extended gesture elicitation to customizable multi-finger interaction using a commercial ring-based wearable device. The work combined gesture elicitation with gesture recognition, showing that user-defined gesture vocabularies can be effectively integrated into practical recognition pipelines.

### 2.4. Wearable Interaction for Smart Homes and IoT

The increasing deployment of IoTs technologies has created new requirements for natural and efficient interaction with interconnected devices [20]. Smart homes now integrate lighting systems, thermostats, multimedia equipment, security systems, robotic devices, and numerous connected appliances [49]. Traditional interaction techniques often require visual attention, explicit device selection, or speech, making them unsuitable in many everyday situations.

Gesture-based commands issued through smart rings can reduce cognitive workload, preserve user mobility, and provide discreet interaction without interrupting ongoing activities [50,51]. The ability to recognize subtle finger gestures [6] further enables interaction in socially sensitive environments where voice interaction is undesirable or impractical.

Despite these advantages, most existing IoT interaction systems primarily emphasize recognition performance and hardware implementation while devoting comparatively little attention to the systematic design of gesture vocabularies. Consequently, identical IoT commands are frequently associated with different gestures across platforms, limiting consistency [24] and increasing users’ learning effort [42].

### 2.5. Research Gap

The literature demonstrates substantial progress in ring-based sensing technologies and gesture recognition algorithms, but three important gaps remain. First, relatively few studies combine ring-based sensing with user-centered gesture elicitation to derive consensus-based interaction vocabularies. Second, most existing research evaluates recognition algorithms using predefined gestures rather than gestures naturally proposed by users. Third, only limited empirical evidence is available regarding gesture consensus, memorability, and usability for smart-home interaction using smart rings. Fourth, we did not find any study involving shared gestures that are expected to simplify home control by allowing anyone to learn common actions from others and to execute them by looking at someone else’s gestures, even without needing a personal profile or device.

To address these limitations, this paper presents Oringano, a framework for designing and evaluating a vocabulary of shared ring-based gestures for controlling IoT devices in a smart home. Gestures are qualified as *shared* in that any gesture performed on a ring—whether it comes from the consensus vocabulary or is a new gesture acquired by a user in context—can be shared from one user to another, provided they belong to the same group. This study adopted a mixed-methods research methodology combining systematic literature analysis, requirements elicitation, gesture elicitation study, prototype implementation, and empirical experimentation. In this way, the proposed approach bridges the gap between advances in ring-based sensing technology and the practical design of intuitive interaction techniques.

This methodology enables the investigation of both technological and human factors involved in ring-based gesture interaction while ensuring that the resulting vocabulary can be implemented using current sensing technologies.

### 2.6. Ethical Considerations

The study was conducted in accordance with institutional ethical guidelines for human-subject research. Participation was voluntary, informed consent was obtained before data collection, and all collected data were anonymized before analysis. Before data collection, the participants were informed of the experiment’s objectives, procedure, and potential risks and signed a GDPR-compliant consent form (see Figure A2 in Appendix B). During data processing, all personally identifiable information (see Figure A3 in Appendix B) was anonymized or removed. Psychological and behavioral data were stored on a local computer with restricted access and used solely for this research. The data are not publicly released without additional anonymization and renewed participant consent. Participants were free to withdraw from the study at any time without consequence. Video recordings and ring-based sensing data were used exclusively for research purposes and stored securely in compliance with applicable data protection regulations. This process and the associated forms have been formally approved by the Commission on Ethics in Research Involving Humans (https://uclouvain.be/en/research-institutes/ipsy/ethics-commission, accessed on 31 August 2026) of the Psychological Sciences Research Institute (IPSY) (https://uclouvain.be/en/research-institutes/ipsy/the-institute, accessed on 31 August 2026) of our Université catholique de Louvain under reference “Project 2022-60”.

## 3. Systematic Literature Review

**Figure 2:**
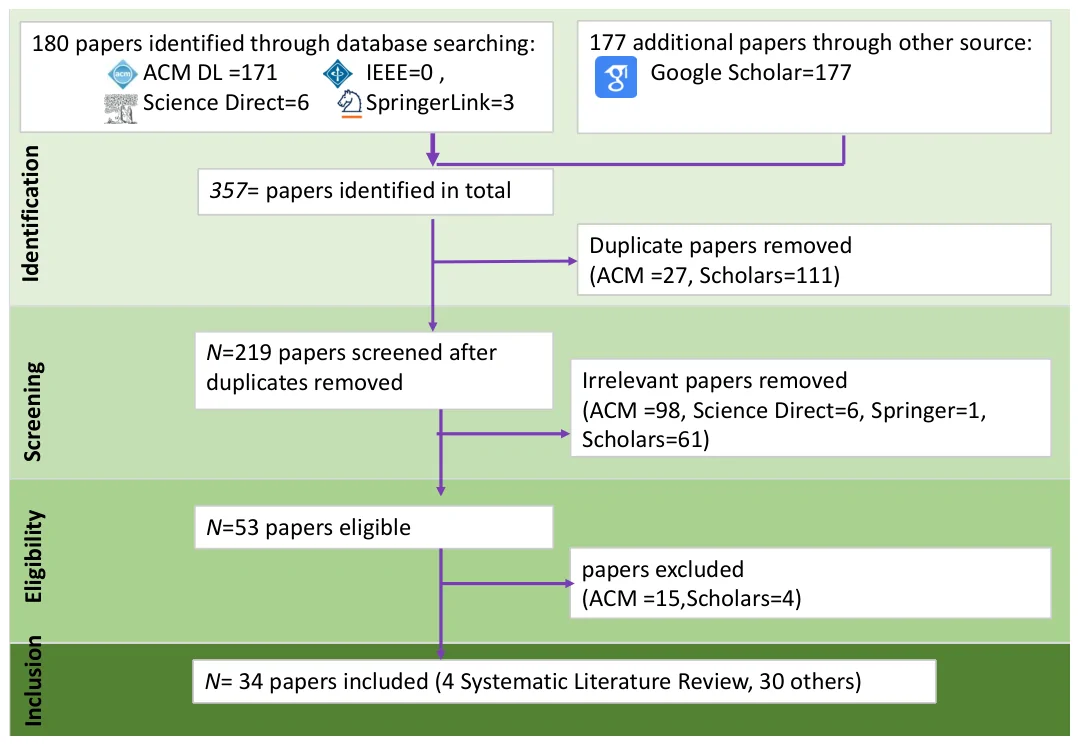
Prisma stages of the SLR.

Since our work investigates ring-based gestures to support collaboration across different systems, we review prior work on ring-based interaction, focusing on how gestures have been conceptualized, implemented, and studied. This section details our Systematic Literature Review (SLR), a procedure that provides a fully structured, exhaustive, and replicable synthesis of all available evidence on a defined topic to ensure transparency and completeness [52]. We outline our method for conducting a Systematic Literature Review (SLR) [53], organized as a four-phase Prisma (Preferred Reporting Items for Systematic Reviews and Meta-Analyses) [54] procedure consisting of Identification, Screening, Eligibility, and Inclusion phases, which are further detailed in the next subsections (Figure 2).

### 3.1. Phase 1/4: Identification

We address three research sub-questions, which collectively contribute to our $RQ_{1}$:

*RQ_SLR_*_1_ What types of gestures have been proposed for ring-based interaction in users’ common environment, such as IoT?
*RQ_SLR_*_2_ What are the current state-of-the-art tools/innovations related to smart rings in mobile computing?
*RQ_SLR_*_3_ How can ring-based gesture input be evaluated in mobile computing?

To define our search strategy in a systematic and reproducible way, we followed the same underlying logic as the PICO (Population–Intervention–Comparison–Outcome) framework [55] for structuring systematic review searches. We adapted this framework for our HCI-focused study on ring-based gestures. This framework ensures comprehensive coverage while maintaining transparency and minimizing bias in query formulation.

- *Population* (P): Gesture elicitation studies investigating ring-based gestures in a smart home, namely, for controlling IoT devices.
- *Intervention* (I): Gestural manipulation of a smart ring, operationalized in the search string through terms describing appropriate reliance on the ring as a device input: (“gesture” AND “smart ring” AND “gesture elicitation”) combined with interaction-related terms (“HCI” OR “Evaluation/study”) AND (“Application mobile”), to capture gestures performed and application contexts (“smart home” AND “IoT”).
- *Comparison* (C): We did not encode a specific comparison condition as a search filter since this is optional.
- *Outcome* (O): Outcome-related terms were excluded from the query and instead used during screening and data extraction. HCI papers often describe systems and interactions without making outcomes explicit in titles or abstracts.

**Figure 3:**
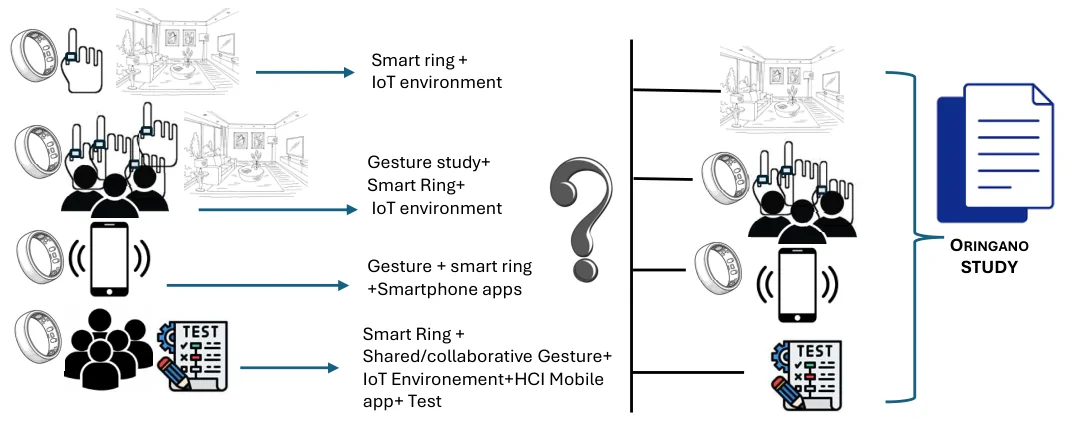
Queries used to position Oringano with respect to related work.

We searched for papers potentially relevant to our topic of investigation using the following query (Figure 3):

Q =[“smart rings”and “hci” and “mobile application” and “evaluation” ] AND [(“IOT”) OR (“gesture” and “elicitation”)].

which we ran in several digital libraries, including both single-publisher libraries (e.g., IEEE Xplore) and multi-publisher engines (e.g., Google Scholar). We selected the following four major Computer Science digital libraries: ACM Digital Library (https://dl.acm.org/search/advanced, accessed on 5 February 2026); IEEE Xplore (http://ieeexplore.ieee.org/, accessed on 5 February 2026); Elsevier ScienceDirect (http://www.sciencedirect.com/, accessed on 5 February 2026); and SpringerLink (https://link.springer.com/, accessed on 5 February 2026).

We also used one multi-publisher source to ensure the completeness and coherence of the references, validate independent query results, and cover other publishers as well: Google Scholar (http://scholar.google.com/, accessed on 5 February 2026). This engine served primarily as verification mechanisms rather than for reference identification; it was used to validate the results obtained from the primary libraries, check for missing relevant papers, and cover publications from other publishers, especially those that are not covered by the aforementioned libraries.

### 3.2. Phase 2/4: Screening

Each paper was evaluated with respect to its relevance to Q using criteria of *form* and *contents*, as follows: *Form.* Qualitative analysis (Classification). We retained only papers written in English that underwent peer review and for which the full text and the resulting gesture set were available. *Contents.* We retained only papers that used ring-based gestures as the primary form of the interface and mentioned user manipulation or gestures in the context of interaction.

### 3.3. Phase 3/4: Eligibility

At this stage, all remaining papers involved ring-based gestures and described user manipulations of gestures. We then assessed whether they provided sufficient information on the outcomes of ring-based gesture interactions to be included in our analyses. We considered ineligible and further excluded those papers that explicitly mentioned a gesture performed with a ring, but did not report the results of the interactions or detailed outcomes of the interaction. This exclusion criterion was necessary because our study focuses on the usability of ring-based gestures in the frame of a smart home.

### 3.4. Phase 4/4: Inclusion

This phase consisted of verifying quantitative and qualitative aspects of our corpus of papers. For the quantitative analysis, we employed the following tools to create the corpus:

1. Zotero 7.0 (https://www.zotero.org/), a multi-platform bibliography management software tool used for managing our corpus. Each reference has been fully imported, including all its metadata. Automated tags were created for all keywords (both internal and external), and a colored tag has been added (e.g., blue for ACM, green for IEEE, gray for SpringerLink).
2. pdf2text-ocr (https://racosa.github.io/pdf2text-ocr/, accessed on 31 August 2026), a software for automatic extraction of text from PDF files. The extracted text was submitted to automatic language processing and analysis.
3. PaperMachines (http://papermachines.org/, accessed on 30 July 2026), a Zotero plug-in for content analysis.

### 3.5. Outcomes

We found four relevant SLRs with different focuses (Table 1). Bilius et al.’s GestuRING [50] explicitly conducts “a systematic literature review of ring-based gesture input” by delivering a dictionary structuring 579 gesture-to-function mappings for ring, ring-like, and ring-ready devices found in the literature. Wang et al.’s SLR [28] is broader as it covers 206 smart-ring publications and splits them into interaction input/output and passive sensing. Villarreal et al.’s studies [33,34] performed a qualitative analysis of gestures found in GESs with any device, not just rings. Some SLRs were excluded because they did not address our primary focus, even though they employed a comprehensive study. For example, Hosseini et al. [56] selected over 100 sources in their SLR according to Prisma 2009 guidelines [57], but they cited smartwatches without mentioning ring-based gestures.

### 3.6. Gesture Elicitation Studies with Smart Rings

Wang et al. [28] define rings for Internet of Things (IoT) as follows: *”Smart Rings are small, circular electronic devices that are designed to be worn on a phalanx of a finger.”* Bilius et al. [50] reported that the first reference of gesture input for rings was presented in 1997 by Fulumato and Tonomura. An analysis of 16 prior ring-based interfaces shows that gesture interaction has become a particularly popular technique in recent designs for interaction acceptability [4,6]. Vatavu et al. [59] provided deeper insights into users’ stroke-gesture articulation behavior, ultimately leading to better-designed gesture sets and more accurate recognizers. It presents a set of gesture accuracy measures about pen pointing, that describe the way gestures unfold and what happens during their production related to their ideal forms. In medicine, Clough et al. [60] defined a gesture as follows: *“Gesture is a fundamental component of language that contributes meaningful and unique information to a spoken message and reflects the speaker’s underlying knowledge and experiences. Theoretical perspectives of speech and gesture propose that they share a common conceptual origin and have a tightly integrated relationship, overlapping in time, meaning, and function to enrich the communicative context.”*, which reveals that a gesture expresses both a function and an asset.

Gheran et al. [4] conducted the first GES investigating user preferences for smart ring-based gestures in 2018. Their analysis of a dataset comprising 672 elicited gestures from 24 participants revealed a low level of inter-user agreement (M=0.112), thereby underscoring the inherent challenges in designing consensus-driven gesture sets. Subsequent work by Herath et al. [12] collected over 3000 gestures and achieved 92.4% accuracy using machine learning techniques for touch-based interactions, while Kurz et al. [9] reported recognition accuracies of up to 98.8% using motion sensor data.

A replication by Gheran et al. [46] analyzed 240 ring-based gestures across five referents and rediscovered only about half of the original gestures, raising concerns about reproducibility. The study identified two limitations: the use of a subset of referents and users limited familiarity with smart rings. The authors therefore emphasize the need for broader replications across devices and contexts, such as in [47]. Villarreal et al. [34] further structured GES research by defining contexts of use, organizing referents, and categorizing gestures based on representational and implementation modalities. Their review also classifies studies across domains such as IoT, mobile computing, and extended reality.

Finally, Wang et al. [28], building on [61], clustered gestures in four categories: within-hand, hand-to-surface, encumbered [40], and mid-air gestures [62,63], further divided into 15 subcategories. Within-hand gestures are the most studied, with 195 instances across 17 references and reported accuracies ranging from 82% to 99%. Additional studies highlight contextual applications: Bardot et al. [40] showed that mid-air and on-ring gestures are more efficient under encumbered conditions, while Yeo et al. [41] demonstrated the potential of combining smartwatch and ring gestures.

### 3.7. Rings as an Alternative or Extended Input

Beyond SLRs of gesture interaction, several studies investigate smart rings within specific platforms and application contexts. Early research on ring-based interaction focused on the diversity of finger movements and gestures performed both on surfaces and in mid-air [4,45]. These gestures were conceptually linked to interaction paradigms such as digital pens and later extended to rings for predefined [32] or user-defined gestures [30].

Beyond mid-air gestures [63,64], smart rings have been explored for everyday interaction tasks. For example, Kim et al. [19] proposed the IRIS system, which combines camera and IMU sensing to enable instance-level control of smart home devices under strict size, weight, and power constraints. In mobile gaming, Cheung et al. [13] demonstrated that users benefit from using a smart ring to adjust map orientation, improving precision in viewing angles. Smart rings have also been investigated as alternatives to traditional input devices. Studies [16,65] proposed virtual keyboard systems based on ring tilt interactions. Notably, the RingVKB system [16] supported up to 48 key inputs, including alphanumeric characters, achieving approximately 93% recognition accuracy.

More recent research situates rings within broader ecosystems, including IoT [12,14,19] and around-device interaction [13]. Smart rings are integrated with IoT platforms and other wearable devices such as smartwatches [41]. Applications span diverse domains, including industrial contexts [40] and healthcare monitoring [11,14]. Smart rings are explored as augmentations to digital peripherals, supporting interaction in scenarios where users’ hands are occupied [15] or encumbered [40].

### 3.8. Smartphones and Smart-Ring Interaction

Early developments in smart ring research include their integration with other devices, such as with another input device [66], with a smartwatch [9,41], with a smartphone [10,19], a drone [67], a television [12], and a keyboard [16]. The integration of smartphones and IoT has since been widely explored [20,68], particularly in mobile interaction contexts. Smart rings paired with smartphones function as complementary input devices, enabling data acquisition and interaction analysis. For example, magnetic sensing techniques allow rings to act as passive input devices by leveraging the smartphone’s magnetometer to detect gesture-related position and orientation without requiring onboard power [13]. Recent systems further demonstrated multimodal interaction capabilities. Kim et al. [19] evaluated the IRIS smart ring with 23 participants, showing that such systems are unlikely to replace voice assistants but rather coexist with them. Similarly, Tang et al. [11] introduced the τ-ring, a multimodal sensing platform connected to a smartphone application for physiological monitoring and handwriting recognition, achieving 88.5% accuracy for letters and 55.0% for words. Finally, novel interaction techniques continue to emerge. For example, Kim et al. [69] proposed BudsID, a finger-identification method enabling smart rings to control earbuds through finger-specific inputs. Building on this body of work, the following section focuses on the use of smart rings for simple, everyday interactions within IoT environments, such as controlling smart TVs and other connected devices.

### 3.9. Ring Interaction in a Smart Home

#### 3.9.1. Smart Rings in a Smart Home Based on SLR

Drawing on the 12 most cited GES studies [34], we selected a subset of relevant references aligned with our research context and updated their citation counts. Table 2 presents the most influential studies—chosen for their relevance to our research questions ($RQ_{1}$–$RQ_{3}$)—alongside additional frequently cited works [64,70,71,72,73] and more recent contributions [47,51]. The latter emphasizes the importance of replication for deriving generalizable gesture sets in smart home contexts.

#### 3.9.2. Smart Rings with Extended Input in a Smart Home

IRIS [19] is the first system that combines a camera and IMU to enable instance-level detection and control of connected home devices while adhering to size-weight-power constraints. IRIS outperforms voice assistants in both user preference and latency. Nonetheless, its usability in smart home settings could be further enhanced by improving gesture-set scalability and boosting detection accuracy for small or distant household objects. Jing et al. [39] proposed MagicRing, a low-memory-footprint approach in which an intermediary device converts RF signals emitted by a ring into IR commands to operate home appliances. While promising for smart home control, this method still requires additional evaluation to confirm its practical effectiveness.

#### 3.9.3. Smart Rings and Smartphone Input in a Smart Home

Several experiments have been conducted, including replications, to validate their findings and studies on ring-based gestures in smart home or/and with smartphones [11,46,50]. Table 3 shows an overview of these experiments. Bilius et al. [8] focused on a specific target population to assess the added value of smart rings for elderly people. Even though smart rings have been available at affordable prices [11] and have evolved with the market, their concept continues to be studied to explore their potential. In our work, we want to check whether any value can be added to the IoT home and leverage the smartphone, the most commonly used tool in daily life, to enhance the capabilities of the smart ring.

## 4. Requirements Elicitation for a Smartphone Application

**Figure 4:**
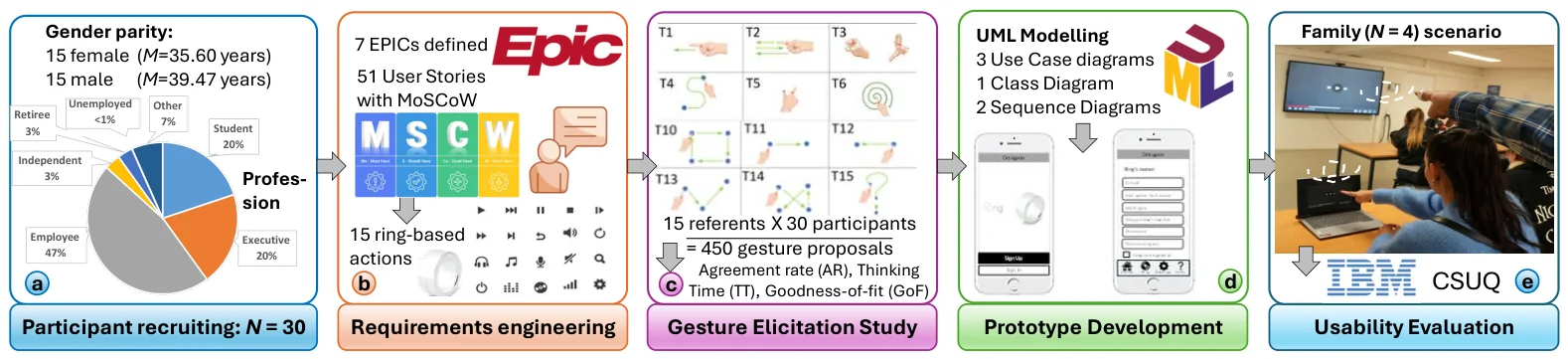
Pipeline of the Oringano research method: (**a**) participant recruiting, (**b**) requirements engineering, (**c**) gesture elicitation study, (**d**) prototype development, and (**e**) usability evaluation.

To address our research questions, we capitalize on the results of our review exploration of ring-based gestures to design a prototype application that assists a smart ring’s user in gesture input with IoT. To this end, Figure 4 describes the five main stages of our research method for investigating ring-based gestures for control actions in a IoT-based smart home, where gestures can be shared among members of a user group. These stages are further developed in the next sections.

### 4.1. Participant Recruiting

**Figure 5:**
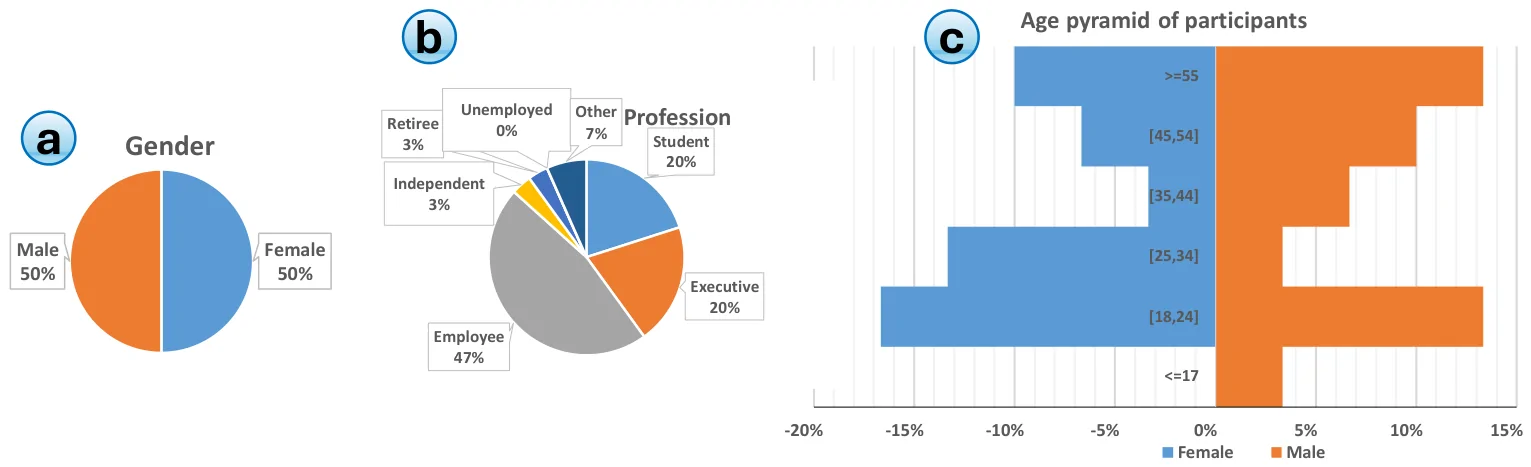
Distribution of sociodemographics of participants: (**a**) gender balance, (**b**) backgrounds, and (**c**) age pyramid.

We recruited thirty (30 participants is a common benchmark from the Central Limit Theorem for certain statistical analyses [77]) independent and voluntary participants (Figure 5a, aged from 14 to 67 years old, *M* = 37.53, *SD* = 15.31, *Mdn* = 33.50) through an internal contact list in different organizations and via social networks. They were selected to preserve gender balance (15 female, *M* = 35.60, and 15 male, *M* = 39.47—see Figure 5a), to cover six age groups (Figure 5c), and different backgrounds and occupations, such as health, banking, IT, and communications (Figure 5b). None of the participants had prior experience with any smart ring, including Logbar’s Ring Zero device (https://vimeo.com/137048667?from=outro-embed, accessed on 30 July 2026) used in our setup, the same device used in other studies [4,45,46,47]. All participants reported moderate to extensive use of desktop computers (*M* = 6.57, *SD* = 1.05 on a 7-point scale), smartphones (*M* = 6.67, *SD* = 0.60), and tablets (*M* = 3.30, *SD* = 2.51). They did not suffer from any disability and had corrected to normal vision.

### 4.2. Approach for Requirements Engineering

**Figure 6:**
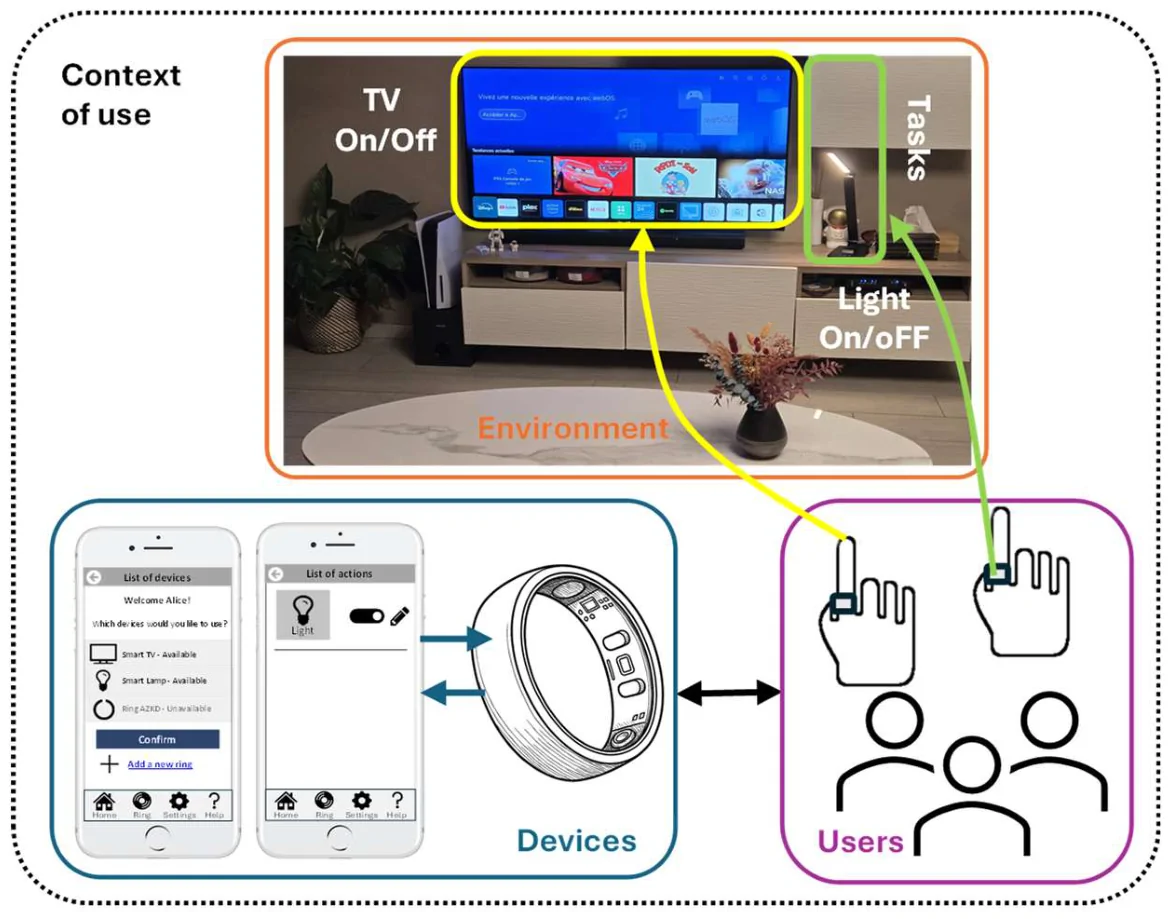
Oringano’s context of use for scenario-based design: one or many co-located users control IoT devices in a smart home using one or many smart rings by shared gestures.

To support scenario-based design [78] in this stage (Figure 4b), we asked participants to consider the following scenario, inspired by the limitations (Section 2): “A mobile application running on a smartphone can be used by one or more users in a family setting, using one or more smart rings to perform gestures that control multiple connected devices (i.e., visible to our application, regardless of whether they are of different types, such as two smart TVs and three smart lamps). These devices and appliances are located in the same room, their models are included in the application database to enable end-to-end communication, and a local network architecture supports a small number of these connected devices and their most common models”. This scenario is therefore materialized in a context of use [3] represented in Figure 6 and communicated to the participants. We collected end-user expressions (An *end-user expression* consists of a simple statement written with a natural language template that describes a scenario from the specific viewpoint of the user interacting with the application). An end-user expression is typically written as “As a [type of user], I want [an action/goal] so that [a benefit/value]”) for this scenario and organized them into 51 user stories [79] clustered into 7 defined epics (An *epic* in agile development methods generally refers to an agile epic, which is a large body of work that can be broken down into smaller, more manageable tasks known as “user stories”). An epic should not be restricted to a single sprint and should address several user stories, such as an important functionality:

1. The technical, usability, and accessibility aspects of the application.
2. The profile management of end users willing to use the features of the application and perform ring-based gestures.
3. The management of connected devices.
4. The management of smart rings and their gesture configurations, including sharing gestures among users and individual personalization of existing gestures mapped to actions.
5. The management of permissions for devices, rings, and features.
6. The supporting management to facilitate user onboarding.
7. The security management to prevent unintended consequences.

User stories are structured and formatted according to a unified template [80]: As *[the WHO]*, I want/want to/need/can/would like *[the WHAT]*, so that *[the WHY]*. In this way, user stories inherently address the three fundamental elements of requirements engineering: WHO wants the functionality, WHAT functionality end-users or stakeholders want the application to provide, and the reason WHY the end-users need the application. These dimensions are materialized by a syntax in the template, like the elements between angle brackets in the following: As a *<role>*, I want *<goal>* so that *<benefit>* [79].

Each user story was also prioritized according to the MoSCoW method [81], where *M* stands for “Must have (Moscow): mandatory requirement” (e.g., US-06 and US-16), *S* stands for “Should have (moScow): high priority requirement” (e.g., US-04), *C* stands for “Could have (mosCow): medium priority requirement”, and *W* stands for “Won’t/Would have (moscoW): low priority requirement”. Each user story is assigned to one of the following types (Table 4) [82]: *domain-oriented* (a high-level requirement describing the application’s objectives and user needs, e.g., US-04), *functional* (a requirement that describes the behavior and information managed by the application’s functionality, e.g., US-06), *non-functional* (a requirement that describes the environment necessary for the application to function properly, e.g., US-16).

An independent group of six HCI-trained investigators different from the co-authors, called the *research team*, collected, wrote, structured, and prioritized the user stories depending on their functional value for ring-based gestural interaction, their relevance for a smart home, and the perceived feasibility of their implementation. The thirty participants were interviewed one by one, and the user stories were consolidated by consensus at the end of each interview. Once all participants had been interviewed, the user stories were finalized so that the next phase would begin one week after.

Finally, we instructed participants to mark the most desired actions in this scenario among a prepared list of frequent IoT actions found in the literature [83,84,85], TV services [12,23], and actions performed by gestures [27,70]. In this way, we created a table of 15 actions (see first column of Table 5) that are controlled by ring-based gestures, representing daily common actions. We created a Microsoft PowerPoint presentation showing a *referent* [31] for each action through a before/after action on a single slide, providing a simplified view of the interaction between cause and effect. These referents will serve as stimuli [31] that are presented by *visual priming*, a frequent practice in the scientific literature [35] to guarantee the quality of a gesture elicitation study [86], which is further elaborated in the next subsection.

## 5. Gesture Elicitation Study

### 5.1. Apparatus

We employed one Logbar Ring Zero (https://www.techinasia.com/ring-zero-new-start-japanese-wearable, accessed on 31 August 2026) device, which participants wore on their index or ring fingers of their dominant hand. The Ring Zero is a wearable gesture-input device that translates precise three-dimensional hand and finger movements into digital commands, allowing users to control smartphones, IoT devices, and applications by making specific gestures in mid-air. Functioning over a wireless personal area network, it integrates 6-axis IMUs containing a 3-axis accelerometer and a 3-axis gyroscope with a sampling rate for continuous 3D coordinate tracking at 50–100 Hz during active states. It reports changes in its orientation to a Bluetooth 4.0 connected device. The ring also features a discrete touch-sensitive pad located on the side of the ring chassis and a touch button that detects short (<2 s) and long presses (>2 s).

### 5.2. Procedure and Task

To address $RQ_{2}$ and $RQ_{3}$, we performed a gesture elicitation study [31] (Figure 4c) based on the 15 actions resulting from the previous stage (Table 5). The study was carried out in a quiet usability laboratory. The participants were welcomed to the setup by an experimenter. They were asked to sign an informed, GDPR-compliant consent form that had been formally approved by the ethics committee of our organization (see Section 2.6). Then, they were provided with information about the GES and its general procedure. They were told they could leave at any time, without justification or consequences. No compensation was offered. They were also asked to fill out a sociodemographic questionnaire (e.g., age, gender, handedness, their use of technologies based on a 7-point Likert scale ranging from 1 = strongly disagree to 7 = strongly agree) and to perform a Motor-skill test [87] consisting of pinching each finger with the thumb several times. The purpose of this test was to verify that each participant was able to control their hand movements and had no limitations. All participants passed this test. Each session implemented the original GES procedure [31,35]: participants were presented with the referents, i.e., actions to ensure the function in each context, for which they proposed a Oringano shared gesture to execute those referents, i.e., gestures that fit referents well, are easy to produce and remember. The participants were instructed to remain as natural as possible. The global order of referents was randomized per participant based on a real random generator based on weather temperatures (http://www.random.org). Each session took approximately 20 min per participant. One researcher welcomed participants and checked the form filling, and another experimenter presented referents, ensuring minimal interference from and among the researchers to mitigate the Hawthorne effect. The dependent variables for our within-subjects design are as follows:

1. Agreement Rate (AR): A real variable representing the agreement among participants to propose a gesture for a referent, calculated by AGATe [35] along with its magnitude (AR ≤0.1 = low, 0.1 < AR ≤0.3 = moderate, 0.3 < AR ≤0.5 = high, 0.5 < AR ≤1 = very high).
2. Thinking Time (TT): A real variable defined as the average time elapsed between the first exposure of the referent and the moment when the participant knew which gesture would be preferred, in seconds [4].
3. Goodness of Fit (GoF): A real variable defined as the average rating from 1 to 10 expressing to what extent the participants thought their gesture was appropriate [4].
4. Memorability of the gesture: An integer variable representing the ease of keeping a gesture in memory for future usage [76], expressed as a 5-point Likert scale [88] (1 = hardest to remember to 5 = easiest to remember).
5. Complexity of the gesture: An integer variable representing the complexity of performing the ring-based gesture [31], expressed as a 5-point Likert scale [88] (1 = easiest to articulate to 5 = hardest to articulate).

### 5.3. Results of the Gesture Elicitation Study

**Figure 7:**
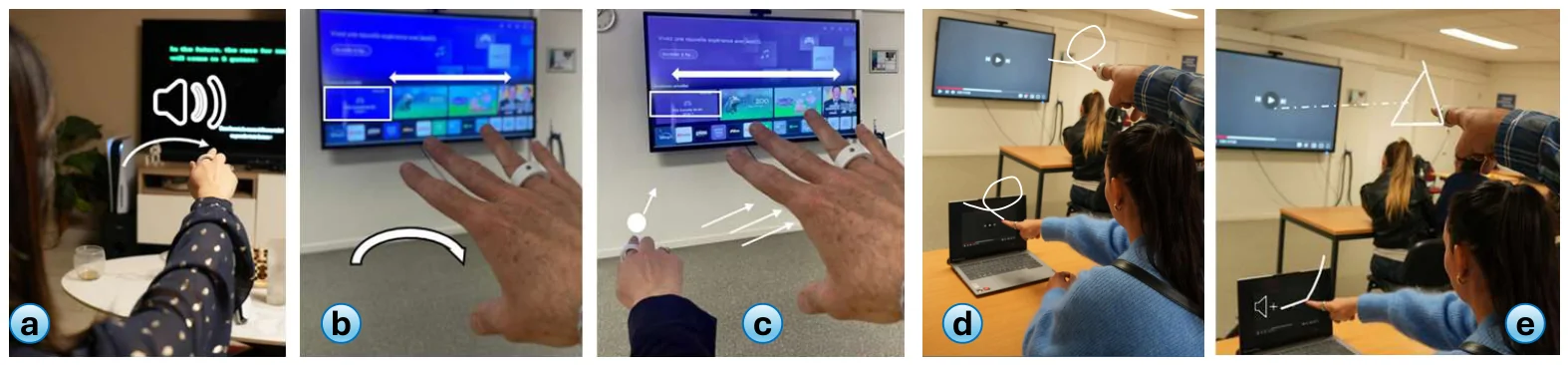
Gestures proposed by participants in our study: (**a**) roll the ring left/right to decrease/increase the volume, (**b**) rotate the hand to the left or to the right by pronation to go to the previous/next item, (**c**) point the index finger forward to play media and move the hand laterally to move within the media, (**d**) roll clockwise one ring to turn the media player on and roll another one counterclockwise to turn it off, and (**e**) draw a symbol in the air to turn the media player on and roll the ring to increase the volume. Arrows indicate directions.

Overall, we collected N=30 participants ×R=1 trial ×P=15 referents = 450 gesture proposals (see Figure 7 for some gestures proposed by participants). Table 5 shows the first (col. 3 with AR) and second (col. 4) most preferred ring-based gestures resulting from the GES. We clustered these gesture proposals by analog gesture types according to the following criteria, the first four criteria being inspired by Bertin’s visual variables [89]:

- *Position*. Gestures issued with a static hand pose (i.e., fingers and hand remain constant), a dynamic hand pose (i.e., the fingers remain constant while the hand is moving in space), or a dynamic hand gesture, i.e., both the fingers and the hand are moving in space) are considered different when they convey different meanings.
- *Size*. Gestures performed with different sizes are considered different depending on the meaning of the size. For example, a large triangle performed with the entire arm is considered different from a small triangle performed with the index.
- *Shape*. Gestures performed with varying shapes are considered different. For example, a triangle is considered different from a rectangle.
- *Orientation*. Gestures performed in different orientations are considered different, e.g., clockwise and counterclockwise triangles drawn in mid-air.
- *Ring action*. Gestures performed with different actions on the ring are considered different. For example, a short press on the button is different from a longer one.

Handedness was not considered in this clustering, as all participants were right-handed and wore the ring on their dominant hand. The research team collected the proposals, applied the criteria, and performed an internal consensus-based coding, rather than an independent coding. When this coding did not reach any consensus, a co-author of this paper served as a moderator to reach inter-rater agreement. As this consensus-based agreement is conceptually distinct from inter-coder reliability based on independent coding [90], the inter-rater reliability of the categorization could not be evaluated.

**Figure 8:**
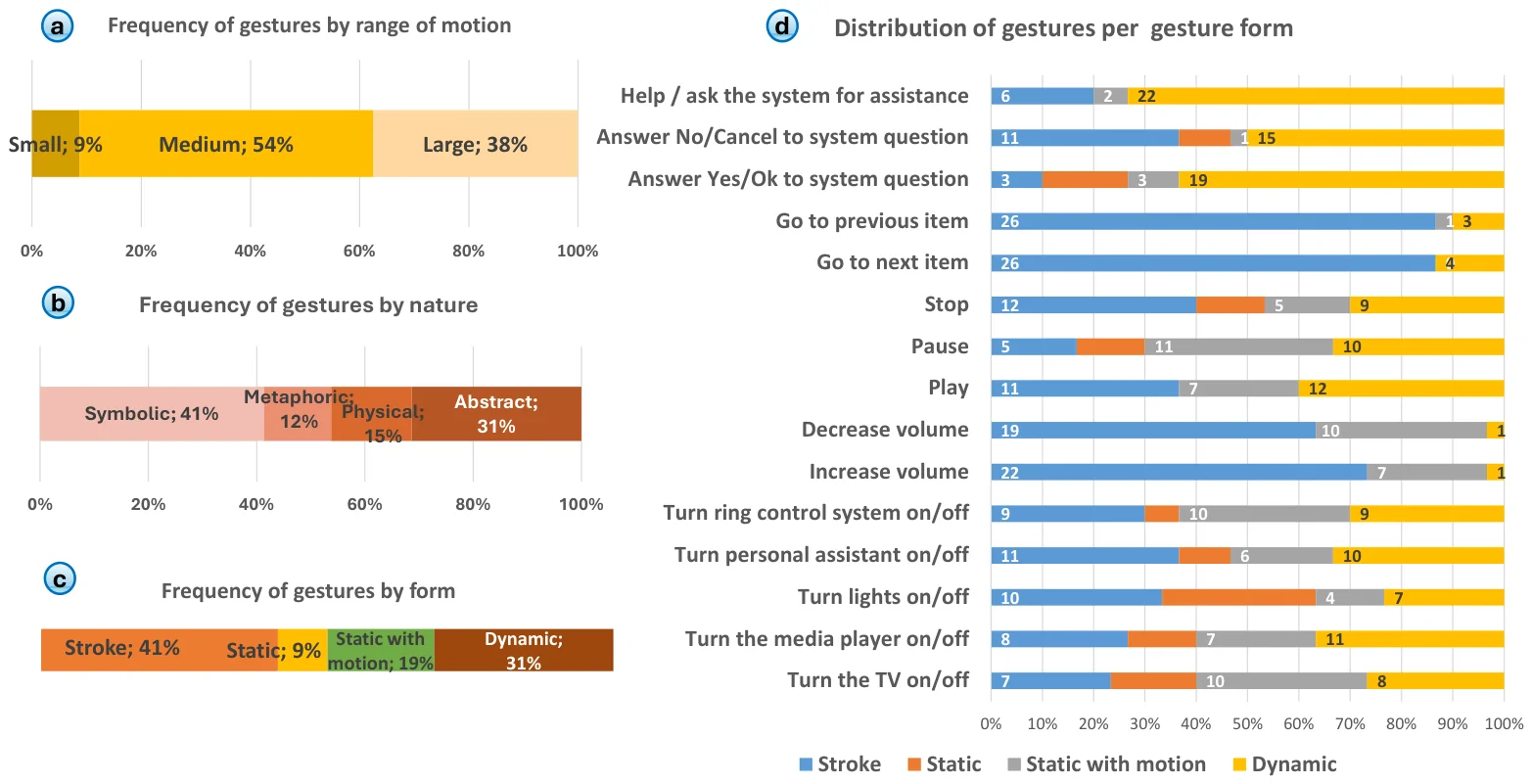
Distribution of gesture frequencies by (**a**) range of motion, (**b**) nature, (**c**) form, (**d**) type.

Figure 8 shows the distribution of the gesture proposals according to four parameters deduced from observing the gestures: *range of motion* (a), *nature* (b), *form* (c), and *type* based on Gheran et al.’s classification [4] (d). Table 6 shows some examples of this categorization. The *nature* describes the meaning of a gesture, with the following categories: *symbolic* gestures depict commonly accepted symbols employed to convey information, such as emblems and cultural gestures; *metaphorical* gestures give shape to an idea or concept [91], such as using the thumb to press a button on an imaginary remote control; *physical* gestures mimic physical actions performed in the real world; and *abstract* gestures have no symbolic or metaphorical connections to their referents. The mapping is conventional. The *form* characterizes the relative importance of hand poses vs. hand motion in the ring-based gesture articulation, with the following categories: *stroke* gestures involve the fingers drawing a trajectory; *static* gestures use a fixed hand pose for some time; *static with motion* gestures keep the hand pose while moving the hand; and *dynamic* gestures combine a changing hand pose and a hand moving. Based on this classification, we observe a high frequency of “Abstract” and “Symbolic” gestures. “Abstract” gestures correspond to those “Draw a letter or a figure” gesture types, while “Symbolic” gestures correspond to “Swipes.” We can also see from this graph that the “Go to next item” and “Go to previous item” gestures are “Symbolic” in over 80% of cases. This is because most of our participants swiped left or right for these two gestures. The distribution across gesture categories reveals consistent patterns across IoT actions [85], such as navigation, media playback, and volume adjustment, where stroke gestures frequently exceeded 60% of usage. Furthermore, actions related to system assistance and mode switching exhibited a more balanced distribution between static and dynamic gestures, indicating task-dependent interaction strategies.

Concerning the *range of motion* (Figure 8a), the results indicate that participants predominantly relied on medium-range gestures (54%), followed by large-range gestures (38%), suggesting that the smart ring should support comfortable and expressive interaction distances in IoT environments. Participants favored ring-based gestures that stay within a comfortable, natural ergonomic space (medium range: 54%) rather than micro-gestures (small range: 9%) or overly expansive full-body reaches (large range: 38%), which may be perceived as tiring gestures. Concerning the *nature* (Figure 8b), symbolic gestures accounted for 41%, reflecting a preference for high-level semantic mappings over purely physical interactions. Participants preferred gestures that rely on universally understood symbols or signs (symbolic: 41%) or that do not have any direct physical (15%) or metaphorical (12%) mappings to their actual functions (abstract: 31%) rather than intuitive, real-world associations. Concerning the *form* (Figure 8c), stroke-based gestures (41%) and dynamic gestures (31%), all together (72%), dominate static gestures (9%) and static with motion (19%), highlighting the importance of continuous motion in smart-ring interactions. Overall, these distributions demonstrate that the smart ring enables a rich and diversified gesture vocabulary, well-suited for both individual and shared IoT interaction scenarios.

#### 5.3.1. Agreement Rate

**Figure 9:**
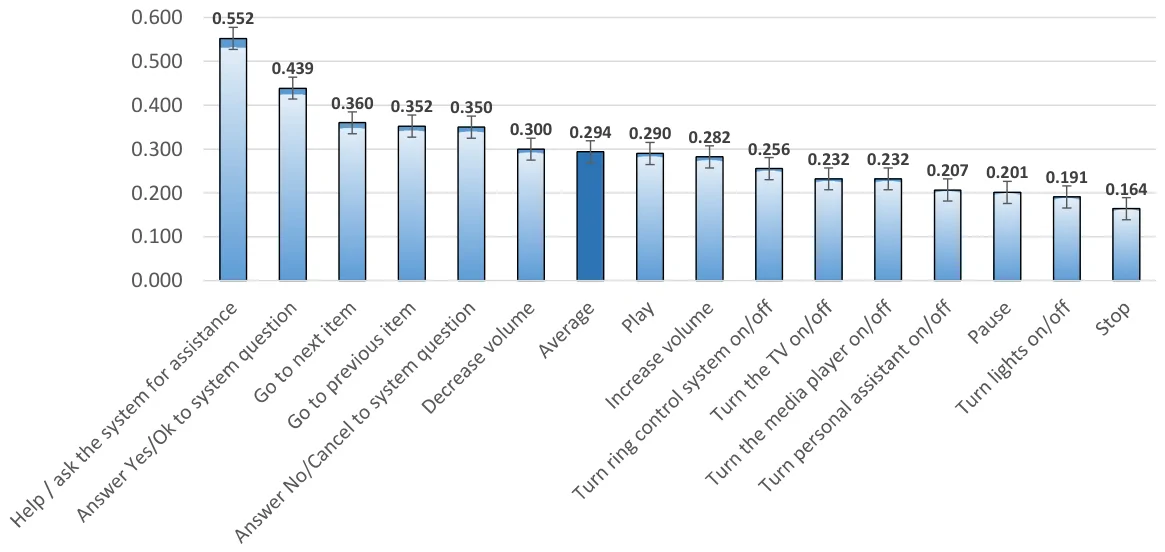
Average Agreement Rate by referent in decreasing order.

Overall, the values (Figure 9) are interpreted as “moderate” to “very high” in magnitude (M=0.29, *SD*=0.10, *Mdn*=0.28), ranging between 0.164 (moderate) for “Stop” and 0.552 (very high) for “Help/ask the system for assistance”. This indicates that participants exhibited acceptable consensus when proposing ring-based gestures, highlighting the inherent variability in user-defined gesture design [51]. Basic actions such as turning devices on/off (e.g., TV: 0.232, lights: 0.191, personal assistant: 0.207) show relatively low agreement In contrast, higher agreement rates are observed for more semantically explicit or contextually meaningful actions. For instance, “Help/ask the system for assistance” achieves the highest agreement (0.552), followed by “Answer Yes/OK” (0.439) and navigation commands such as “Go to next item” (0.36) and “Go to previous item” (0.352). Volume control (“Increase”: 0.282; “Decrease”: 0.301) and playback (“Play”: 0.294) fall closer to the average, indicating moderate consensus. The relatively higher agreement for these actions suggests that participants may rely on more intuitive or culturally shared metaphors for communicative or directional actions. In global sampling, $\frac{9}{15}\;=\;60\%$ of the rates belong to the moderate consensus category and $\frac{4}{15}\;=\;26\%$ of them belong to the high range, and $\frac{1}{15}\;=\;6\%$ belong to the very high range, thereby making them appropriate and eligible for gesture interaction. Most gestures received agreement rates slightly higher than, or close to, those reported in the GES literature (e.g., [35], which summarized 18 agreement rates).

#### 5.3.2. Thinking Time

**Figure 10:**
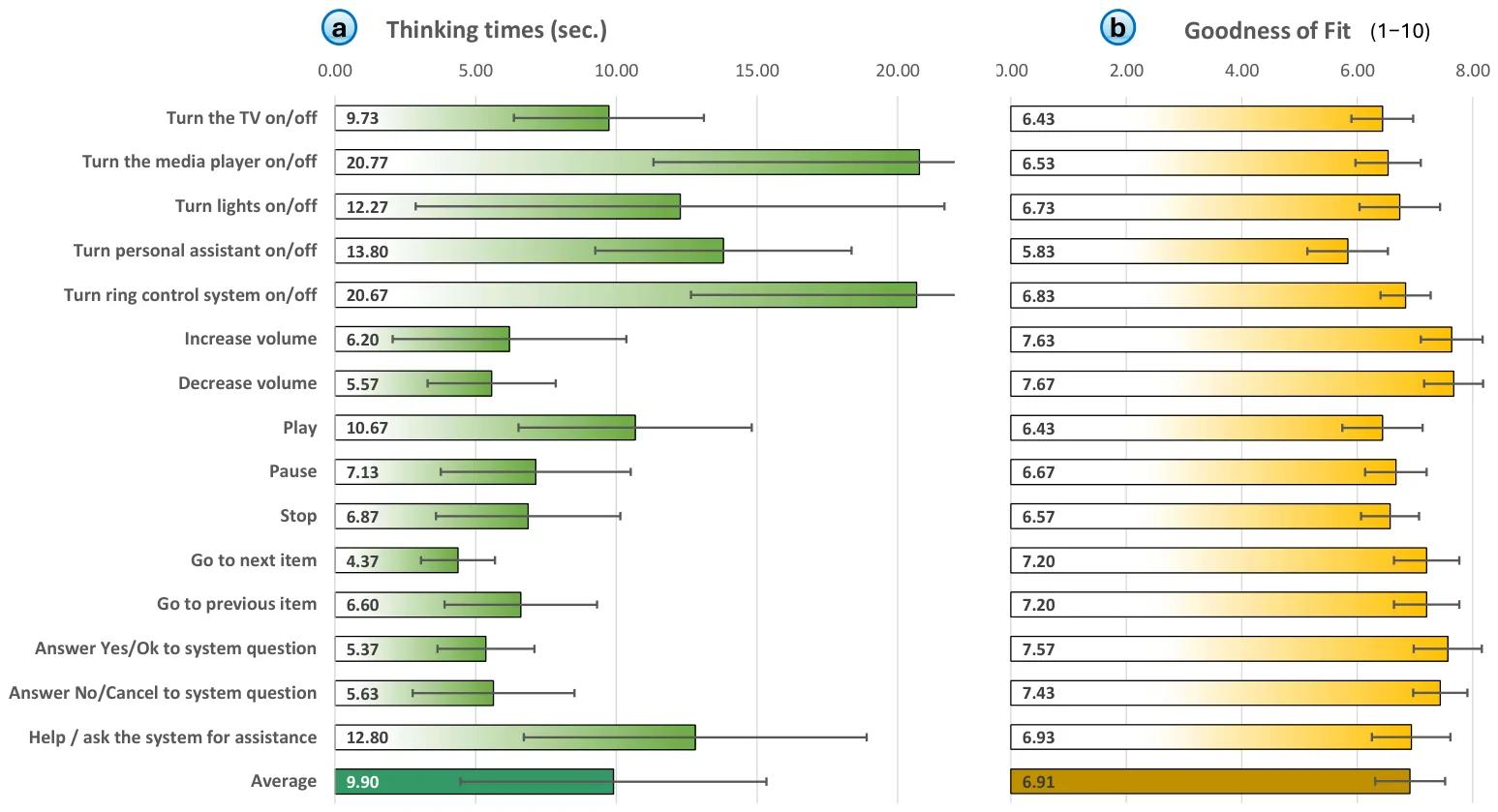
Thinking Times (**a**) and Goodness of Fit (**b**) of shared ring-based gestures proposed for each referent. Error bars show a confidence interval of 95%.

Figure 10a shows the average Thinking Time of ring-based gestures proposed for each referent for the 15 actions. The analysis of this metric indicates notable variability across actions, with both task complexity and familiarity influencing user response. Simple and highly intuitive actions, such as “Go to next item” (M=4.37 s, SD=3.68), and binary responses like “Yes/OK” (M=5.37 s, SD=4.81) and “No/Cancel” (M=5.63 s, SD=8.04), exhibit the shortest times, suggesting that these actions rely on well-established mental models and require minimal cognitive effort. Similarly, basic actions such as “Increase” (M=6.2 s) and “Decrease volume” (M=5.57 s) show relatively low times, reinforcing their intuitive nature. In contrast, more complex or less clearly defined actions, particularly those involving system-level control, such as “Turn the media player on/off” (M=20.77 s, SD=26.40) and “Turn ring control system on/off” (M=20.67 s, SD=22.40), require longer deliberation, indicating higher cognitive load and possible uncertainty in gesture proposition. Moderate times are obtained for everyday actions, such as “Turn lights on/off” (M=12.27 s) and “Turn personal assistant on/off” (M=13.8 s), suggesting partial familiarity but less standardized mappings.

#### 5.3.3. Goodness of Fit

**Figure 11:**
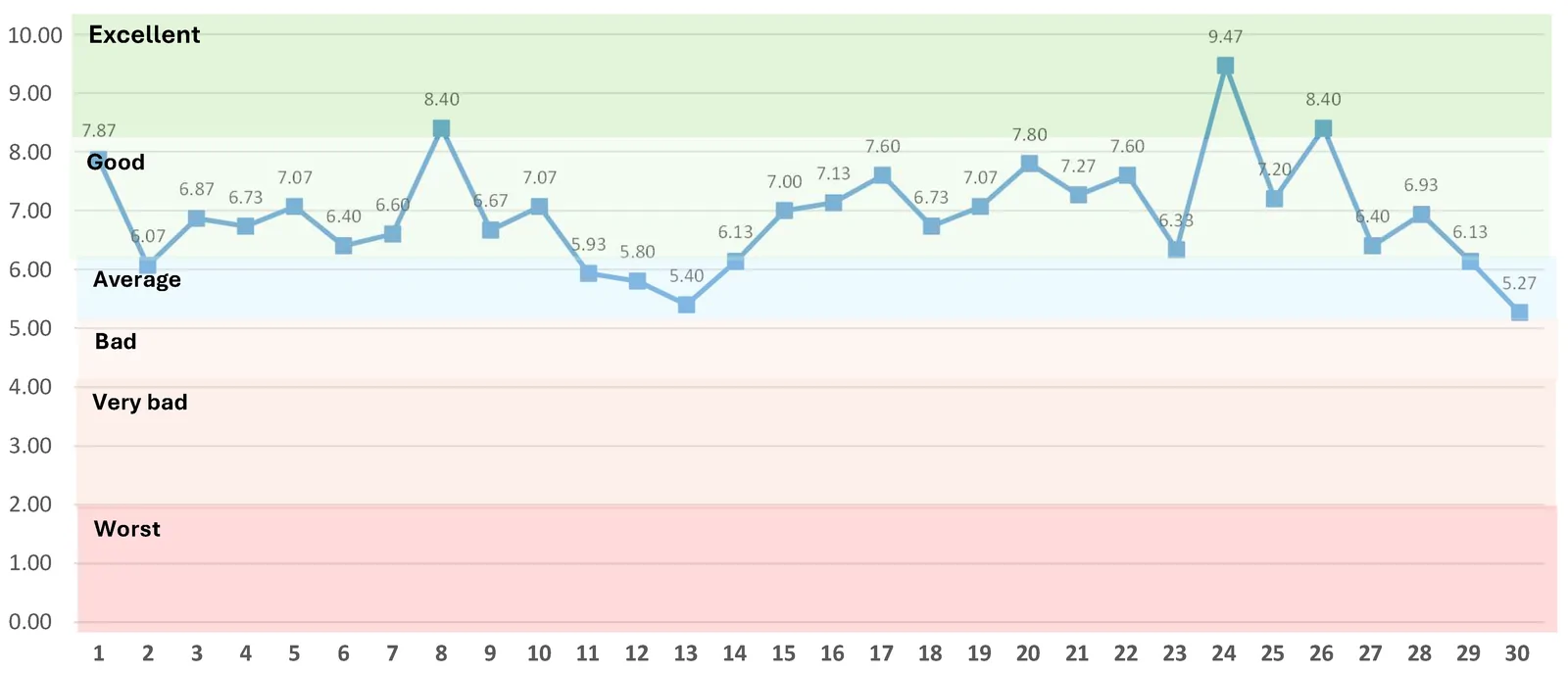
Average Goodness of Fit by participant (P1 to P30).

After proposing a gesture for each referent, participants were asked to reflect on their choice and indicate how well they felt the gesture matched the intended referent. This assessment of Goodness of Fit was self-reported using a 10-point scale, ranging from 1 (low or poor fit) to 10 (high or excellent fit), and served as an expression of participants’ confidence in the appropriateness of their proposals. Figure 10b shows the results for all referents, whereas Figure 11 shows the results for each participant.

Overall, participants perceived the proposed gesture as moderately to highly appropriate (M=6.91, SD=1.69), with average ratings ranging from 5.83 to 7.67. These values are slightly below the average Goodness of fit, where 10 is the best fit, which typically ranges between 7.0 and 8.2, with overall aggregated benchmarks frequently clustering around 7.6 to 7.9 [47], but not significantly (W=20026.5, p=0.25, *n.s.*). This average score indicates that participants generally feel a moderate subjective confidence that their proposed gestures appropriately match the intended referents, which weer expected to be familiar. Gesture mappings for navigation and continuous adjustment tasks received the strongest support. “Decrease volume” obtained the highest mean score (M=7.67, SD=1.42, $\Delta_{95}\;=\;0.51$), closely followed by “Increase volume” (M=7.63, SD=1.49, $\Delta_{95}\;=\;0.53$). Similarly, “Go to next item” and “Go to previous item” were both evaluated positively (M=7.20, SD=1.58, $\Delta_{95}\;=\;0.57$). The narrow confidence intervals associated with these actions further indicate strong agreement among participants regarding the intuitiveness of spatial or directional gestures for navigation and incremental adjustments. System confirmation gestures were also rated favorably. “Answer Yes/OK” achieved a high mean rating (M=7.57, SD=1.65, $\Delta_{95}\;=\;0.59$), while “Answer No/Cancel” received a slightly lower but still positive evaluation (M=7.43, SD=1.31, $\Delta_{95}\;=\;0.47$). These results suggest that confirmatory actions are readily expressible through ring-based gestures, though negative or “Cancel” actions may be perceived as less iconic.

Mid-range scores were observed for binary on/off commands. For example, “Turn the TV on/off” score (M=6.43, SD=1.50, $\Delta_{95}\;=\;0.54$), “Turn the media player on/off” (M=6.53, SD=1.59, $\Delta_{95}\;=\;0.57$), and “Turn lights on/off” (M=6.73, SD=1.95, $\Delta_{95}=0.70$). Although these ratings indicate general acceptability, the relatively large confidence intervals indicate more variability in perceived appropriateness among participants.

The lowest score was obtained for “Turn personal assistant on/off” (M=5.83, SD=1.95, $\Delta_{95}\;=\;0.70$), indicating weaker consensus and reduced perceived intuitiveness compared to other referents. This may reflect the abstract nature of virtual actions, which lack strong physical metaphors and therefore may not induce natural mappings to gestures. Media control actions, such as “Play”, “Pause”, and “Stop”, exhibited consistent evaluations (between 6.43 and 6.67) and confidence intervals ranging from 0.50 to 0.70. These results indicate moderate appropriateness, though not as strong as for navigation or volume control. For example, “Pause” scored (M=6.67, SD=1.49, $\Delta_{95}\;=\;0.53$), and “Stop” obtained (M=6.57, SD=1.41, $\Delta_{95}\;=\;0.50$), suggesting moderately robust participant agreement.

Finally, “Help/ask the system for assistance” received a moderately high rating (M=6.93, SD=1.90, $\Delta_{95}\;=\;0.68$), though its larger confidence interval indicates variation, potentially due to the ambiguity of this referent in everyday smart-home contexts. Across all actions, the combination of positive means and narrow confidence intervals indicates that participants generally found the consensus gesture set coherent and suitable for the smart ring. The results particularly highlight strong fit for directional, continuous, and confirmation gestures, while suggesting opportunities for refinement in system toggles and assistant-related actions.

#### 5.3.4. Other Measures and Comparison

**Figure 12:**
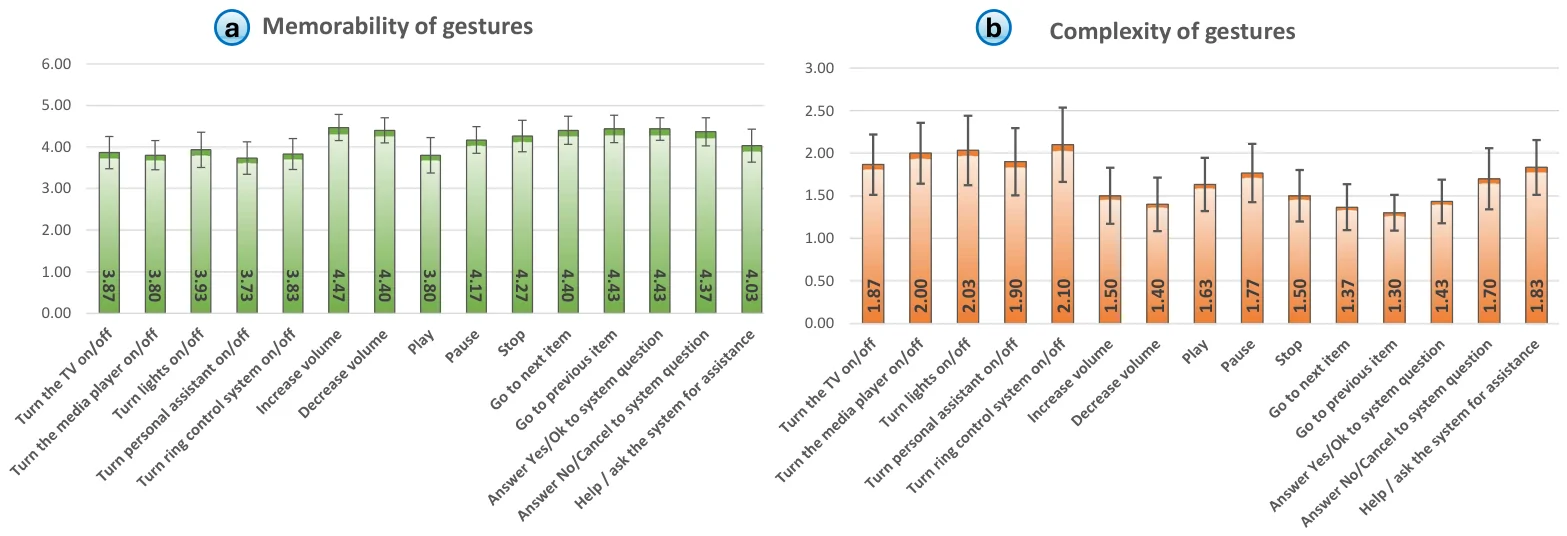
Evaluation of the gesture Memorability (**a**) and Complexity (**b**). Error bars show a confidence interval of 95%.

Figure 12a shows the average Memorability (1–5) of ring-based gestures proposed by participants: the list of gestures suggested for collaboration/sharing is easy to memorize, with the lowest score of 3.7/5, is significantly above the median of 3 (W= 63,400.5, p≤0.001 ***), with a maximum of 4.7/5, which is close to 5. Figure 12b shows the average Complexity (1–5) of ring-based gestures proposed by participants: the scores were also relatively low, given that for each referent, the average is well below that, with a complexity of 2.10 for the action of “Turn off the ring”, whereas the least difficult and most memorable action is “Increase the volume”.

**Figure 13:**
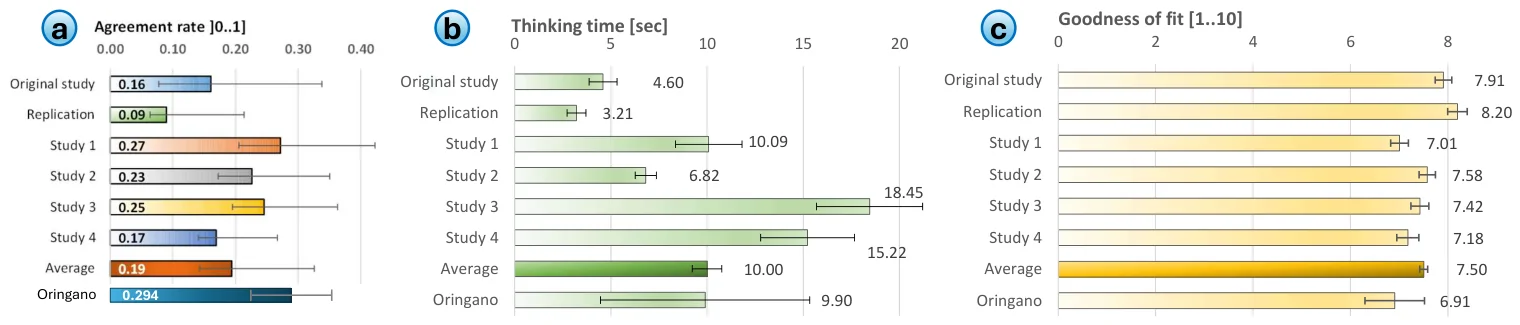
Comparison of the original study [4], a previously published replication [92], four replications [47], and our study in terms of Agreement Rate (**a**), Thinking Time (**b**), and Goodness of Fit (**c**). Error bars show 95% confidence intervals.

Now that we have analyzed the various metrics expressing the quality of the proposed ring-based gestures, let us compare their average results against those of other replications that are not aimed at collaborative, shared manners. We restrict this comparison to only those actions that are common to these GESs (Table 7). Figure 13-left compares the mean Agreement Rates: *AR* = 0.16 in the original study [4], AR = 0.09 in the previous replication [46], AR = 0.228 for four replication [47], compared to AR = 0.272 in our GES, which is slightly higher than the other rates compared to 0.194 for average of all GESs. Results from *t*-tests on independent samples showed limited significant differences, with one difference emerging between the original GES [4] and our Oringano GES ($p\;=\;\;0.027^{*}$), associated with a small effect size (r=0.36) across all referents.

Significant differences emerged across the replication studies. Notably, in the original study, the “Turn TV on/off” action yielded the shortest Thinking Time: 4.2 s compared to 9.73 s in our study. The shortest Thinking Time in our GES is “Go to the next item” (4.37 s), which aligns with the original study (Figure 13). In this figure, we observe differences between the gestures proposed for individual use (as reported with the same measurements in other studies) and those for shared use in our study. Without performing an inferential analysis, we notice that shared gestures as proposed in our study benefit from a higher average Agreement Rate (+84%), a slightly lower Goodness of Fit (−12.64%)), and a longer Thinking Time (+115.2%).

## 6. Prototype Development

In the pipeline of our research method (see Figure 4), this section is based on the prototype development step. The section focuses on the system design and UML modeling: class diagram, sequence diagram, and process architecture.

### 6.1. UML Diagrams of Oringano

**Figure 14:**
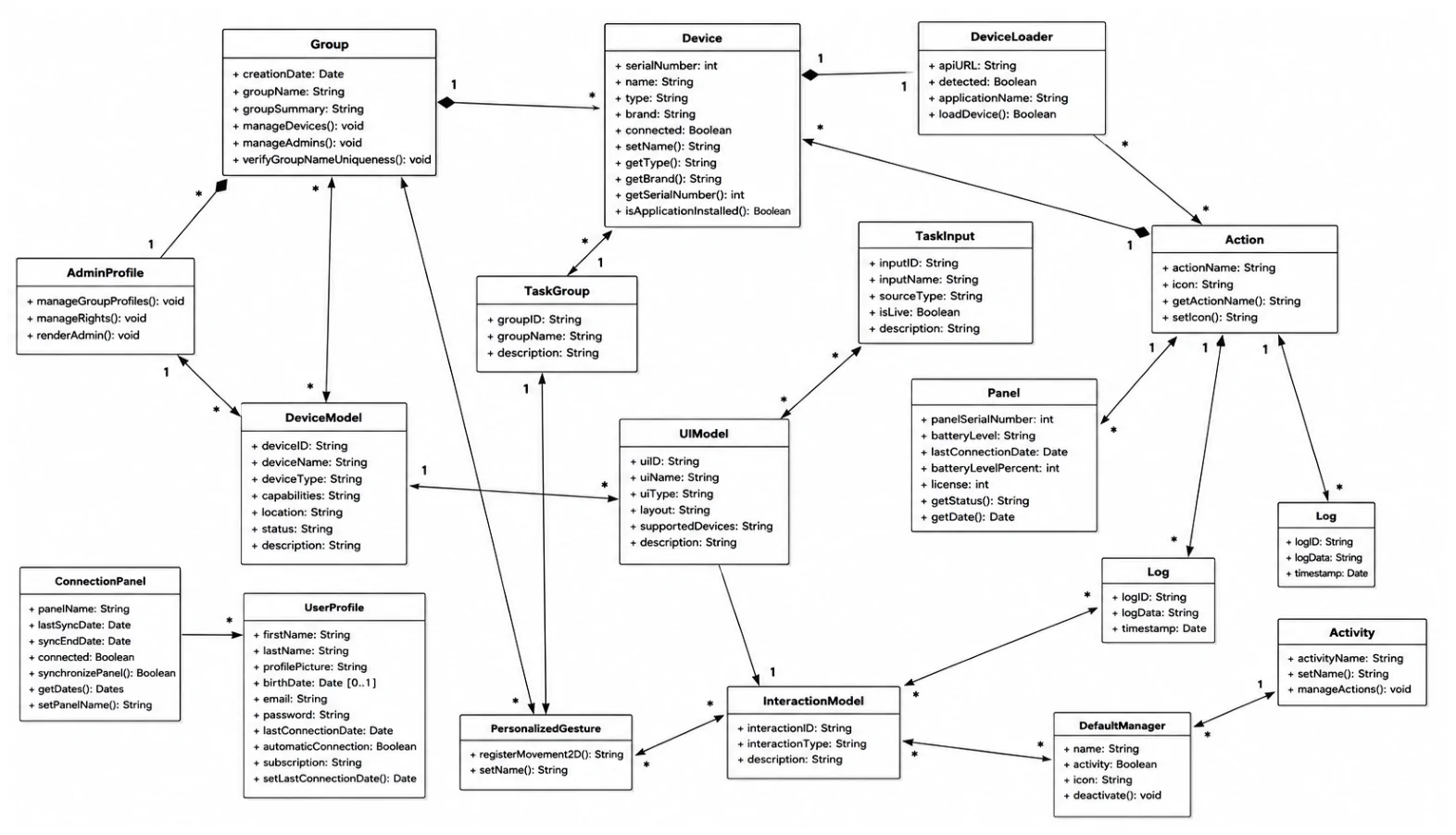
The UML diagram of the Oringano platform to demonstrate the original way with smart rings: the shared gestures with an extension input to set them. * = many.

To design Oringano and better understand how Oringano differs from others [4,46,47], we provided a diagram in standard UML notation to justify the prototype’s design. The structure is represented as a UML V2.5 class diagram (Figure 14). Our application differs from others in its focus on customization of gestures in a group setting. The class Group represents a list that contains profiles and devices, enabling centralized management of devices and access rights. A description of its attributes, methods, and relationships is presented in Table 8.

These specifications are partially aligned with Geeng and Roesner’s study [93], which identified four chronological points during smart home implementation and usage for multi-user interactions: (1) device selection and installation, (2) regular device usage, (3) when things go wrong, and (4) over the long term. Our scenario and its design decisions cover two points as follows: (1) The AdminProfile is the user who has the rights to select and install a ring, while the regular profiles can only join the group; only the administrator can delete the group; the administrator grants access rights to any member. (2) Regular ring usage is ensured for any member. Points (3) and (4) are left unaddressed.

We also have a class containing the default gestures listed in our GES. Specifically, we extract the most frequent gestures for each referent from our GES and derive a default list of gestures for each of the fifteen referents. In addition to creating a group to organize and share personalized gestures, the added value of our gesture diagram lies in the default gesture classes, which are linked to the custom gesture class. This allows users to select their own gesture if they want and make it available to the group (more details are presented in Table 9).

### 6.2. Cloud Integration

**Figure 15:**
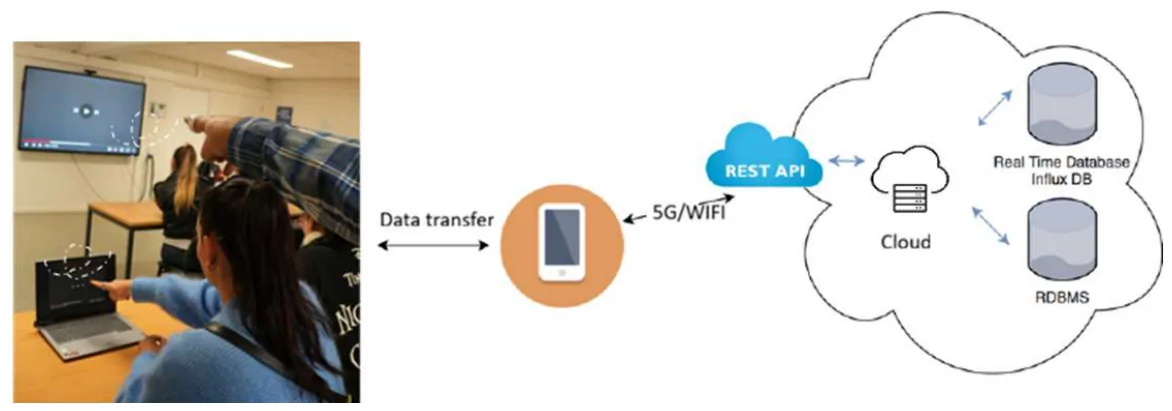
Gesture-based smart ring system with mobile application and cloud integration.

The diagram of Figure 15 specifies a gesture-based interaction system in which a smart ring captures user gestures and stores the resulting data in a mobile application. The collected data are transmitted from the mobile device via 5G or Wi-Fi using an API. A cloud infrastructure provides scalable processing and data management capabilities. The data are persisted in both a time-series database for real-time analysis and a relational database for structured storage and long-term use.

### 6.3. Interaction Sequence Diagram

**Figure 16:**
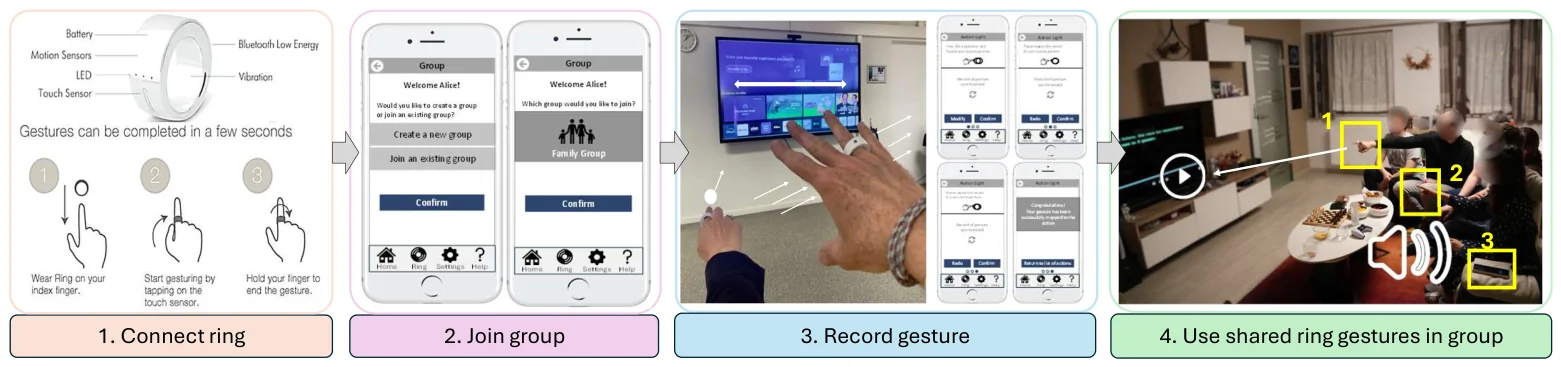
Process view to join “Group” or set for shared gesture and use in IoT environment.

**Figure A1:**
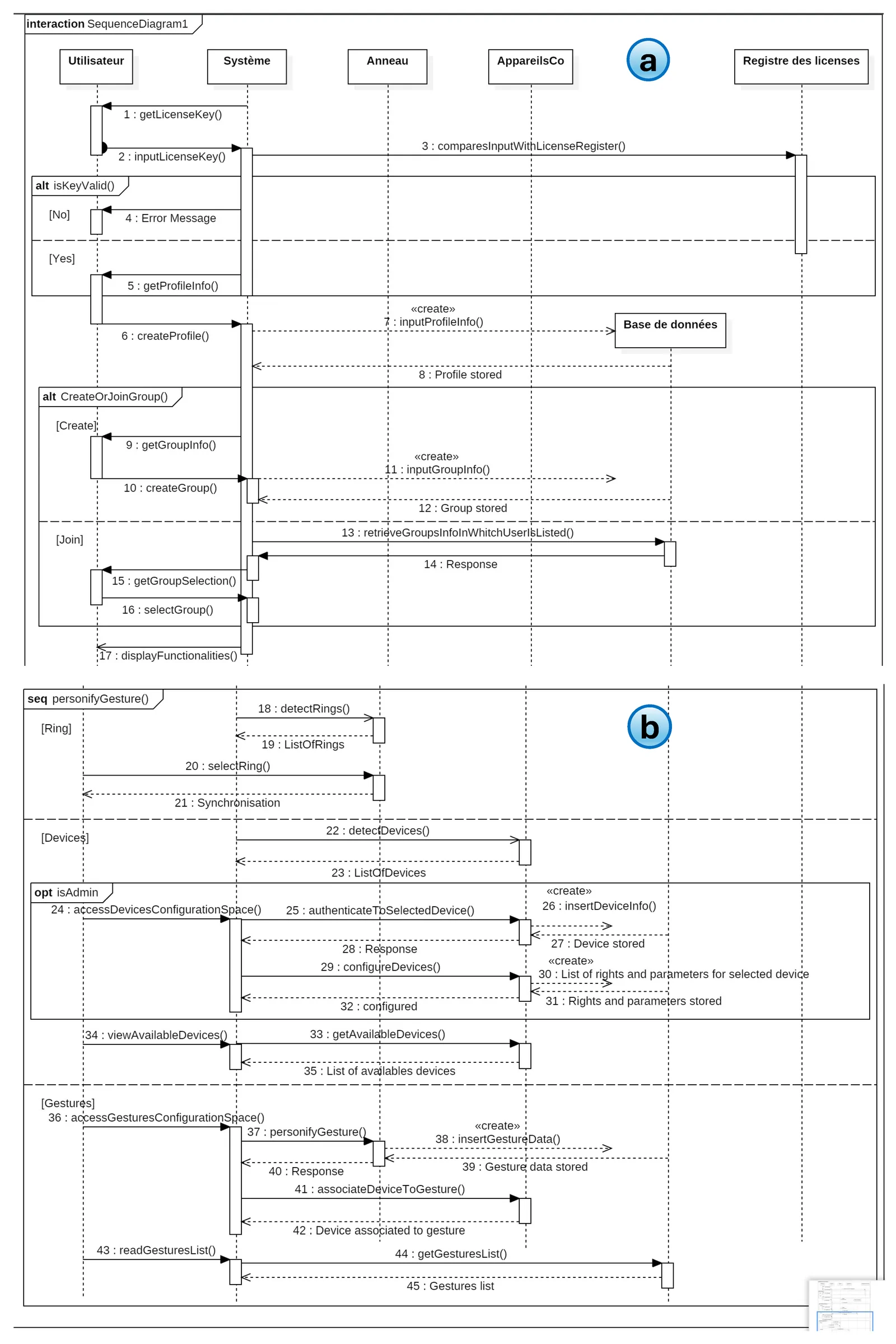
Sequence diagrams of a smart-ring interaction connected with its mobile application Oringano and related to IoT tasks: (**a**) User onboarding; validation and user management; profile registration and group enrollment. (**b**) Customization of gestures and configuration devices.

**Figure A2:**
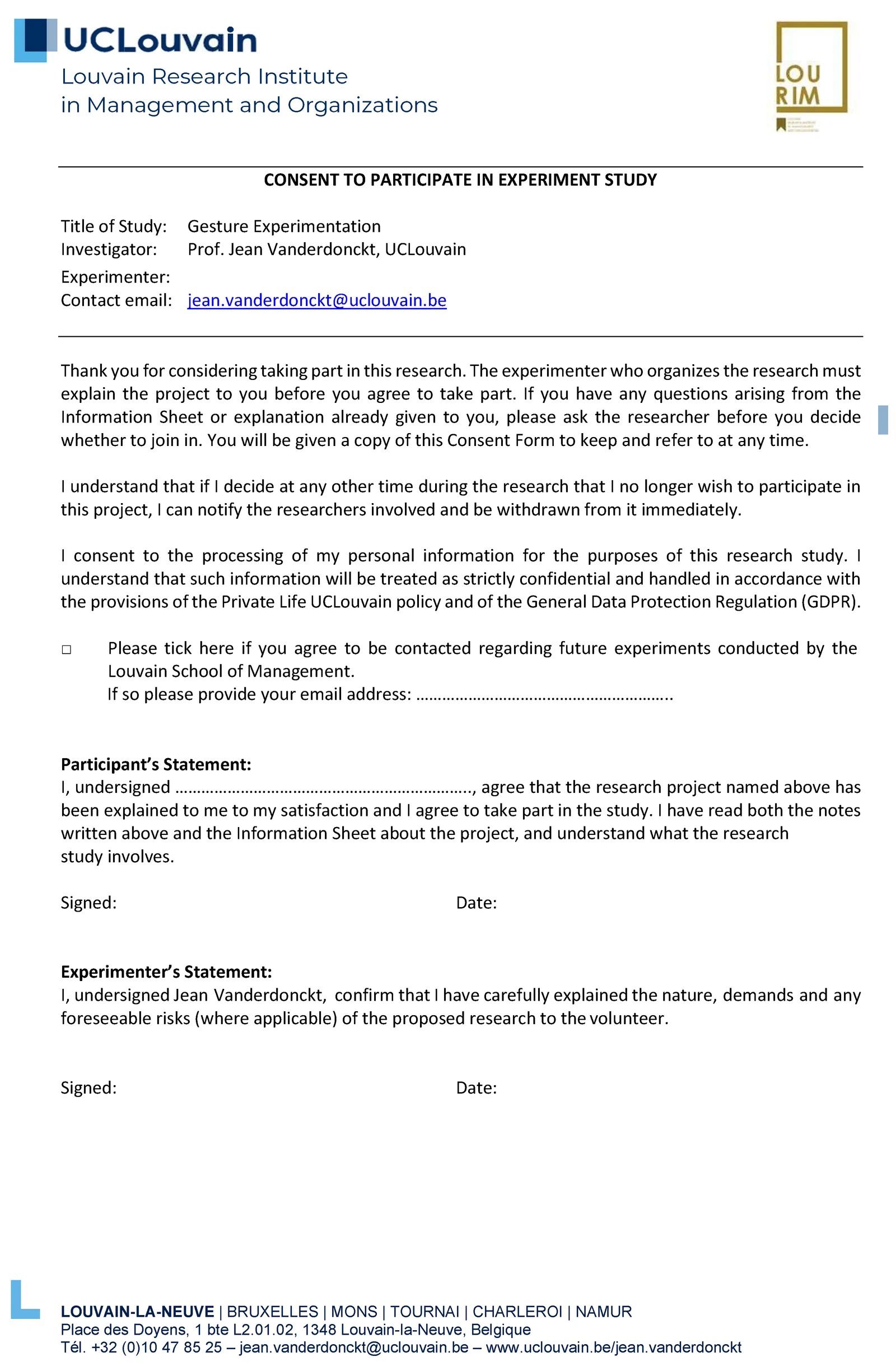
Consent form used in the experiment.

**Figure A3:**
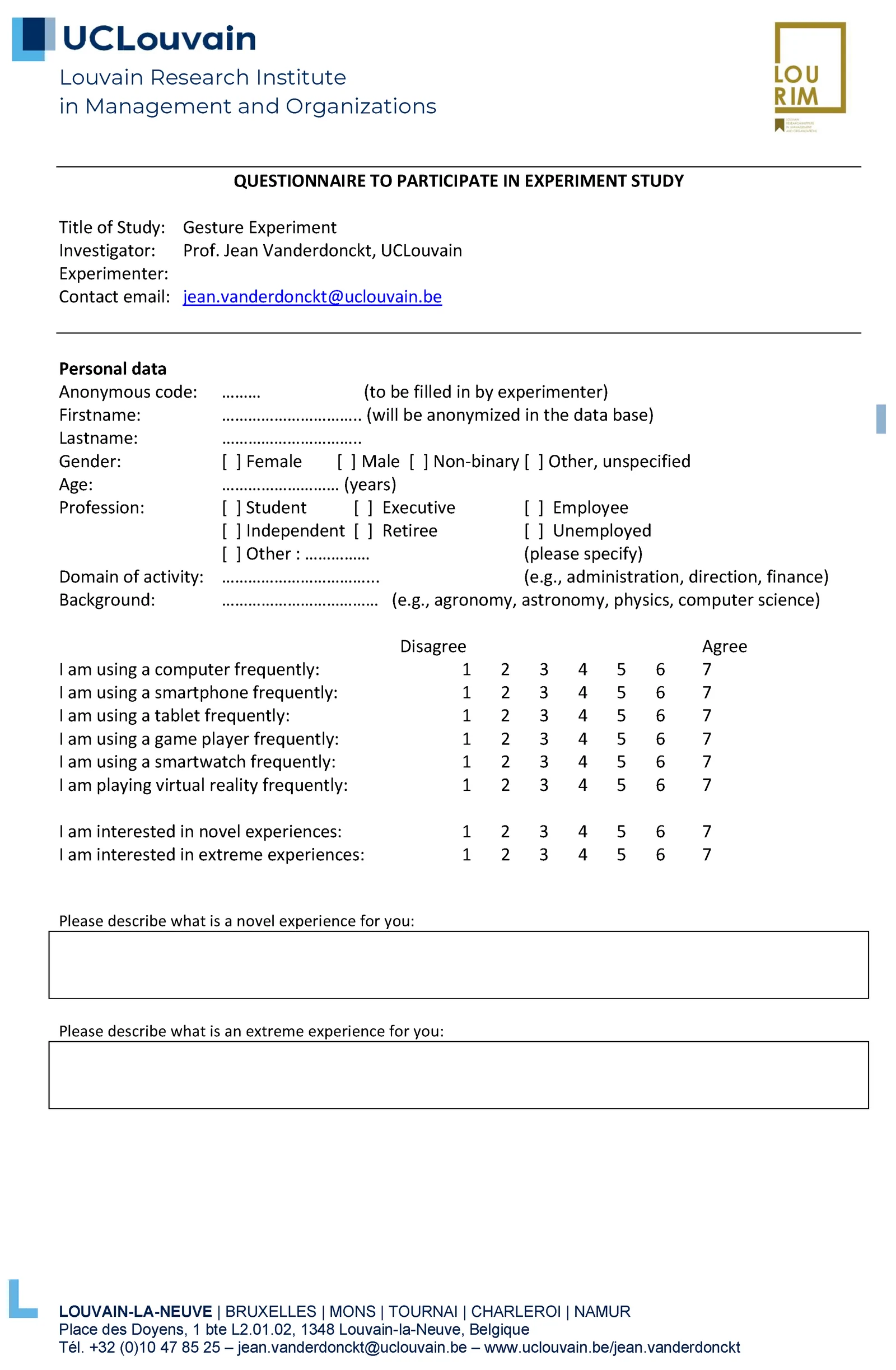
Demographic form used in the experiment.

While the previous diagram describes the overall architecture of the smart ring system and its data flow across components, it does not capture the temporal ordering of interactions. To complement this structural view, the sequence diagram (Figure A1) focuses on the dynamic behavior of the system. It details how gesture data are captured by the smart ring, processed by the mobile application, transmitted to the cloud services, and finally stored in the respective databases, highlighting the sequence of messages and interactions among system components. The presented architecture and its corresponding sequence diagram are directly derived from the defined user stories (Table 4), ensuring alignment between user requirements and system behavior. To illustrate this diagram sequence, we have also designed the process using a more explicit visualization (Figure 16). Based on a user journey map model (see Appendix E), this illustrates each step of the user’s journey, from the moment they activate the smart ring until they complete their task.

### 6.4. User Interface Prototyping

**Figure 17:**
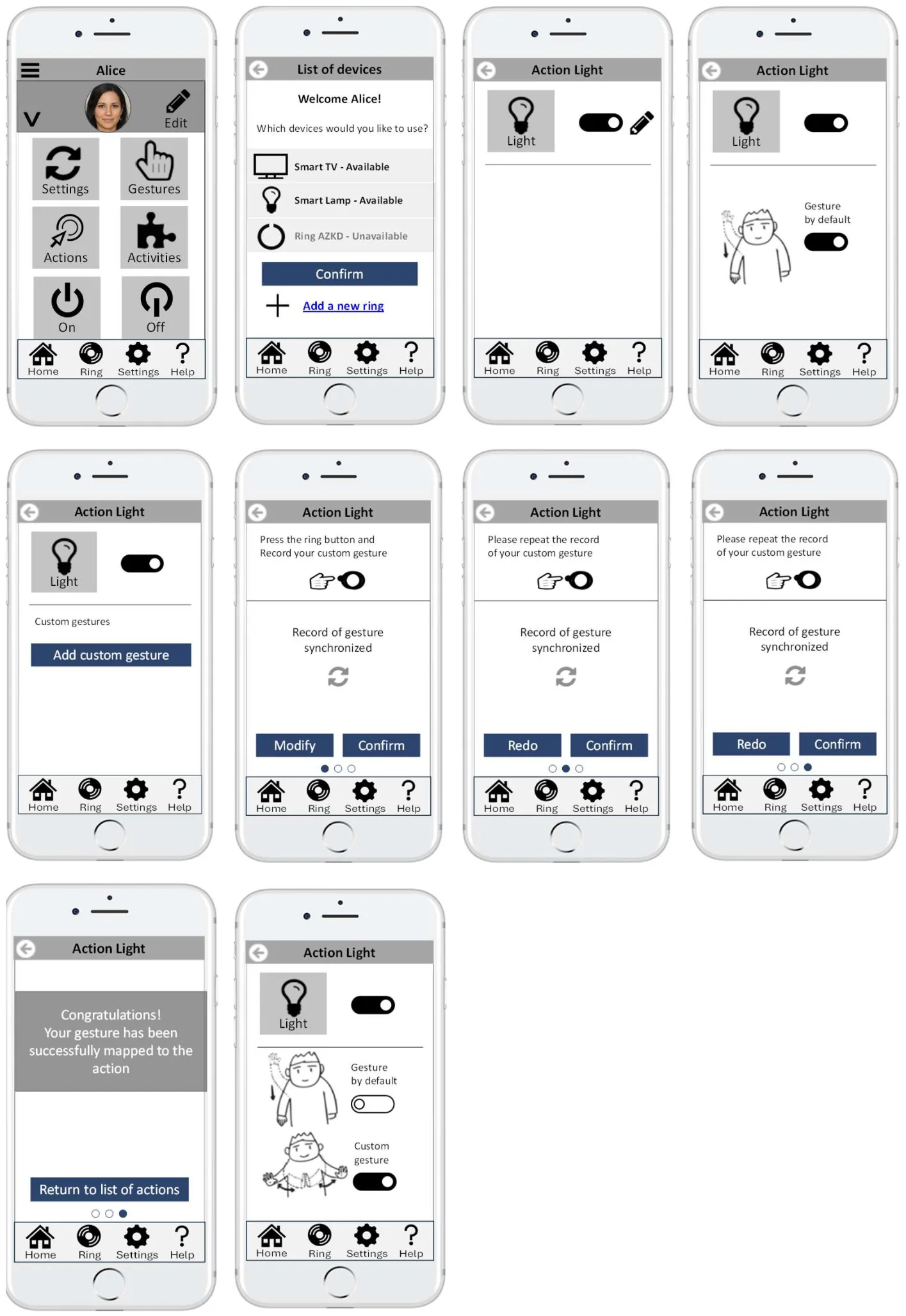
Screenshots for the shared-gesture record for an IoT task with a smart ring in Oringano.

**Figure A4:**
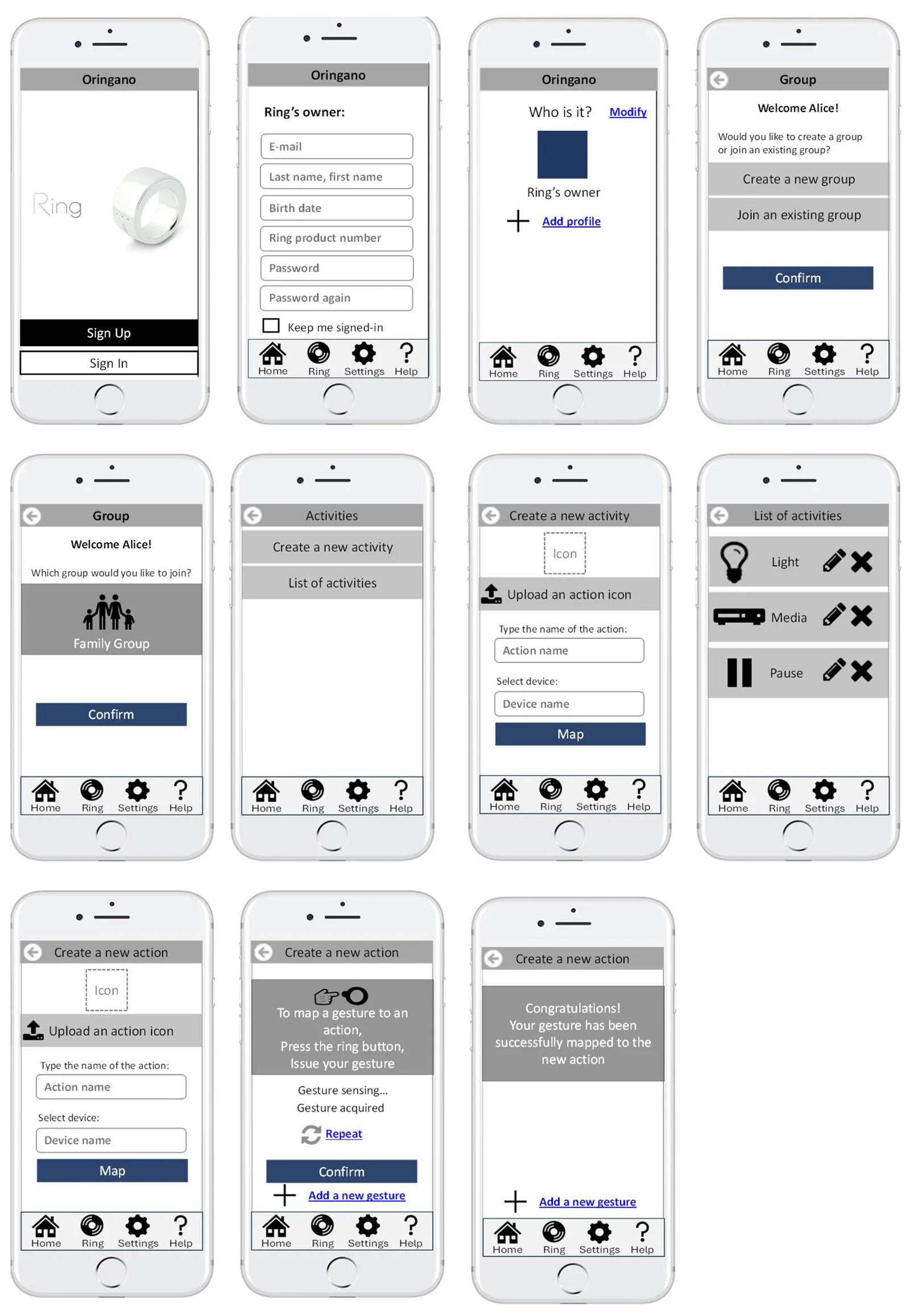
Screenshots of the functions: Scan nearby rings and list metadata. Connect and display device details. Create or join a group/family and modify the name if the user wants.

The prototype enables users to intuitively configure actions and record gestures using the smart ring (Figure 17). It provides a clear workflow in which users can associate a specific gesture with a predefined action through a dedicated configuration screen [36]. During gesture recording, the interface offers real-time feedback to guide users and ensure accurate capture of motion data. To share its recorded gesture with a group, the user must create or join the group with a login connection (Figure A4).

**Figure A5:**
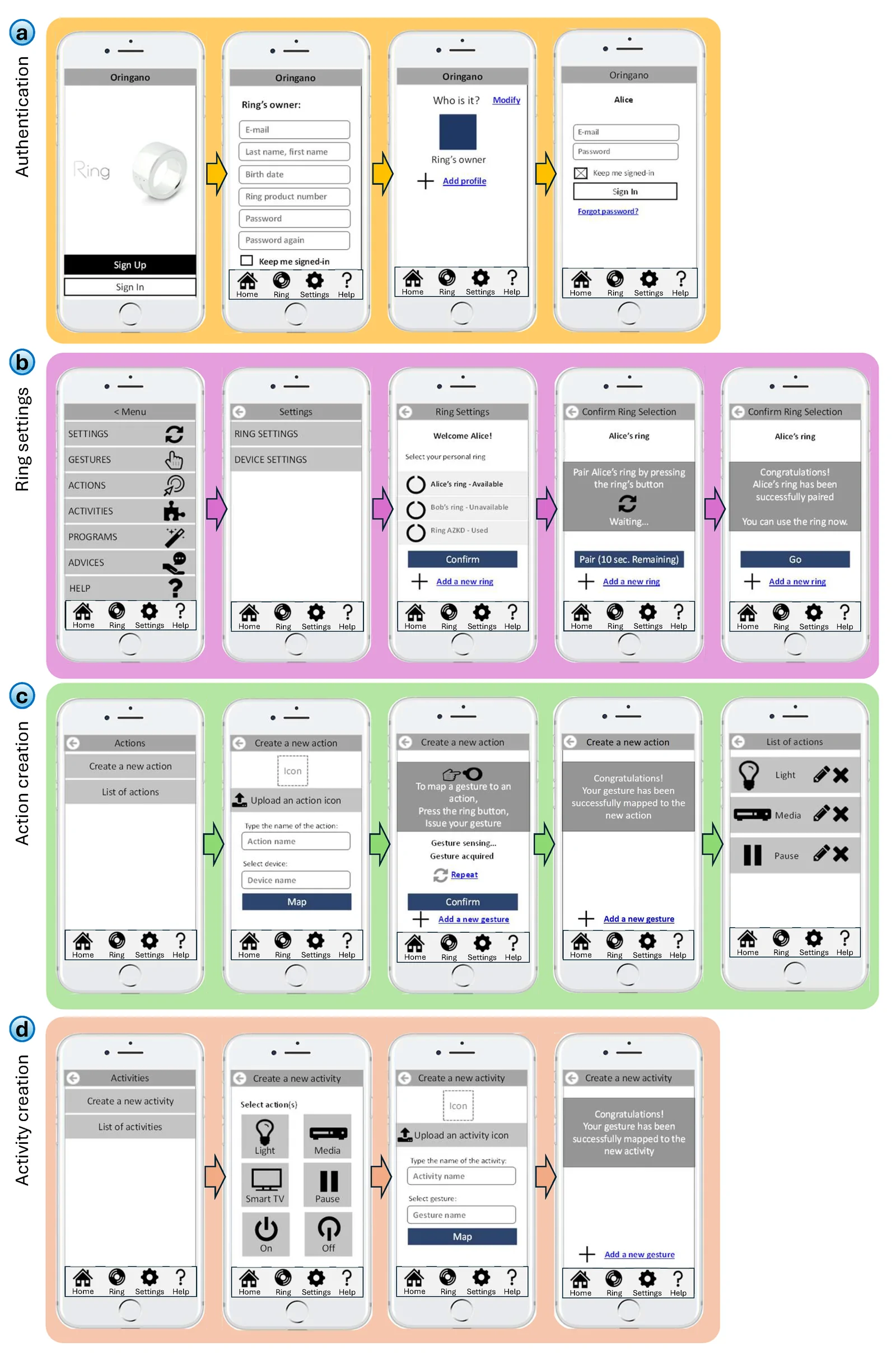
Interaction map of Oringano: (**a**) Authentication or sign in to the app. (**b**) Configure, list, Smart Ring Association by user’s profile. (**c**) Action creation or view the list of actions, such as “Turn on light”. (**d**) An activity allows you to trigger a sequence of actions.

This prototype served as an initial validation of the interaction flow and interface structure. Many tasks, such as authentication (Figure A5a), ring registration and configuration (Figure A5b), gesture records for IoT tasks (Figure A4), shared gestures for a (created) group (Figure 17) in activities and actions, are described in Figure A5c and Figure A5d, respectively. These original features (action and activity tasks) of Oringano are that users can customize gestures for a group of people, which Oringano suggests using a single gesture that combines a set of actions (such as turning down the volume and turning off the TV) into a single activity that the user can create and name [94].

Breve et al. [95] proposed mechanisms that allow regular users to understand and manage actions on security-sensitive IoT devices, such as smart door locks, garage door openers, and smart boilers. Unlike non-critical IoT devices, such as smart coffee makers, connected robotic vacuums, smart light bulbs, and weather stations, as we considered in our scenario, security-critical actions should be accompanied by confirmation gestures, safeguards, or additional validation.

Building on these design artifacts, usability testing was conducted to evaluate how effectively end users can configure actions and record gestures with the smart ring in real usage scenarios.

Regular users join a Oringano group through an email invitation sent by the administrator (Figure 16(2) and Figure 17), which represents a simplistic authentication process (Figure A4). Prange et al.’s study [17] indicates that shared smart-home devices, such as the rings considered in Oringano, create unique privacy and control challenges for multiple users. Some asymmetries between ring administrators and regular users or bystanders may occur. Since the administrator is the person who set up the ring, this person holds primary access rights, which may impose social tensions with other users. We did not investigate these issues further. What we observed, though, is that some regular users are willing to impose their own gestures on the group to become the reference gestures. This option appeared, but was left aside because the gestures customized by each regular user remain personal.

## 7. Experimental Evaluation

### 7.1. Method

The 30 participants were invited to evaluate the usability of Oringano by following a common scenario (see Appendix C for details). To illustrate the evaluation process, participants were presented with a representative user scenario reflecting a typical family use case of the application. The usability study included four tasks:

- Creating a personal profile and configuring user settings.
- Creating a group, adding members, connecting devices, and assigning gestures to actions.
- Creating an activity that combines several actions into a single gesture-triggered sequence.
- Joining an existing group as a guest user. Full task descriptions and transcripts are available in Appendix C.

Each participant signed a consent form and then browsed the aforementioned scenario with Oringano. After selecting three stands, the visitor was asked to fill out an IBM Computer Satisfaction Usability Questionnaire (CSUQ) [96]. This 19-item questionnaire (Table 10) has been empirically validated with a large number of participants using a wide spectrum of interactive systems [97]. It is widely applicable to any interactive system, it is very reliable (α=0.89) [97]. Each item [96] is a closed question measured using a 7-point Likert [88] scale (1 = strongly disagree, 2 = largely disagree, 3 = disagree, 4 = neutral, 5 = agree, 6 = largely agree, 7 = strongly agree). We define the Average-Item-Rating as the mean of participants’ ratings to each item and the following metrics are aggregated: *system usefulness* (SysUse = Average-Item-Rating for items 1–8), *quality of the information* (InfoQual = Average-Item-Rating for items 9–15), *quality of the interaction* (InterQual = Average-Item-Rating for items 16–18), and *system quality* (Overall = Average-Item-Rating for items 1–19). The CSUQ was selected due to its strong psychometric properties, with coefficient alpha values exceeding 0.89 across all subscales. A seven-point Likert [88] format (1 = strongly disagree, 7 = strongly agree) was used, as it allows for three levels of both positive and negative evaluation. In addition to this quantitative assessment, a qualitative component was included through two open-ended questions inviting participants to describe the most positive and most negative aspects of their experience. Together, the standardized questionnaire and the open-ended questions form a semi-structured study design, enabling both quantitative measurement and qualitative insight into participants’ perceptions. The participants were asked to fill out an IBM CSUQ usability questionnaire (quantitative part) and were asked two typical open questions (qualitative part) [98]:

- What did you like the most in the evaluation?
- What did you hate the most in the evaluation?

**Table 10:** IBM CSUQ items used in the usability evaluation of Oringano.

#	Item
1	Overall, I am satisfied with how easy it is to use this system.
2	It is simple to use this system.
3	I can effectively complete my work using this system.
4	I am able to complete my work quickly using this system.
5	I am able to efficiently complete my work using this system.
6	I feel comfortable using this system.
7	It was easy to learn to use this system.
8	I believe I became productive quickly using this system.
9	The system gives error messages that clearly tell me how to fix problems.
10	Whenever I make a mistake using the system, I recover easily and quickly.
11	The information (such as on-line help, on-screen messages, and other documentation) provided with this system is clear.
12	It is easy to find the information I need.
13	The information provided with this system is easy to understand.
14	The information is effective in helping me complete my work.
15	The organization of information on the system screens is clear.
16	The interface of this system is pleasant.
17	I like using the interface of this system.
18	This system has all the functions and capabilities I expect it to have.
19	Overall, I am satisfied with this system.

### 7.2. Results and Discussion of the Usability Evaluation

**Figure 18:**
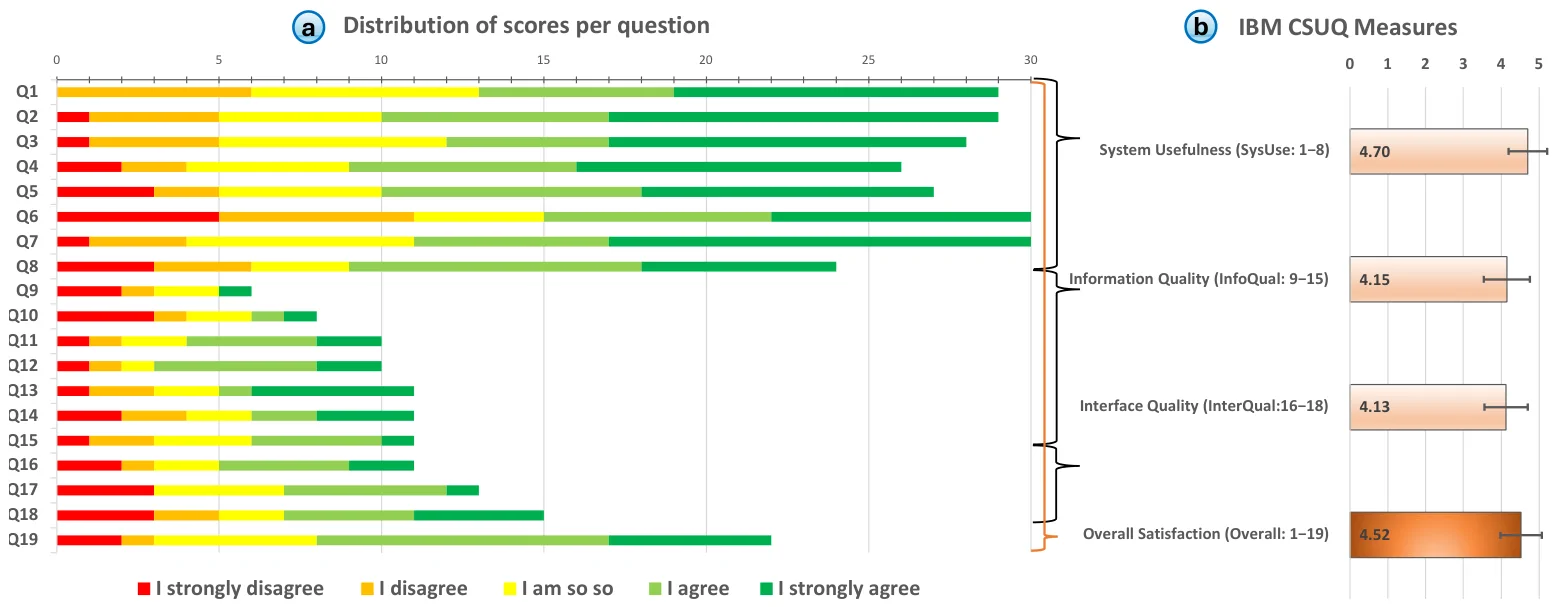
Distribution of scores per question for all participants in each category (**a**); aggregated CSUQ metrics (**b**). Some participants did not answer some questions considered either similar or not appropriate.

The researcher responsible for administering the questionnaires ensured that they were complete and accurate. The questionnaires from all 30 participants were therefore analyzed. When a participant felt that a question was not relevant, they marked it as “N/A = not appropriate,” sometimes with confirmation from the administrator when they asked for it. Figure 18 shows that the results of the CSUQ evaluation, for each of the four metrics, were scored positively above 4.1 (the smallest average score in this evaluation) and well above average, with a slightly higher average for Usefulness (M=4.7, SD=1.43). This suggests that the general attitude of participants regarding the whole process supported by Oringano could be interpreted as satisfying with respect to the goal. Overall satisfaction also yielded a relatively high mean (M=4.52, SD=1.52), indicating a generally favorable overall experience with the application. In contrast, Information Quality (M=4.15, SD=1.70) and Interface Quality (M=4.13, SD=1.60) were rated slightly lower, though still above the neutral midpoint, pointing to potential areas for improvement related to clarity and interface design. The relatively narrow 95% confidence intervals (ranging from 0.51 to 0.61) suggest reasonable consistency in the mean scores. In sum, these results indicate an overall acceptable level of usability, with strengths in perceived usefulness and general satisfaction, alongside opportunities to enhance information presentation and interface quality. Figure 18 also illustrates the distribution of participants’ responses for the 19 CSUQ items. Overall, the distributions show a predominantly positive pattern, with most items receiving a high proportion of agree and strongly agree responses.

Items related to ease of use and learnability (Q1–Q8) were particularly well rated. Most participants reported being satisfied with how easy the system was to use (Q1) and considered it simple and comfortable to operate (Q2 = “It is simple to use this system”: M=4.9, Mdn=5, SD=1.89, Q6 = “I feel comfortable using this system”: M=4.3, Mdn=4.5, SD=1.94). Indeed the whole process supported by Oringano in shared gesture system could be interpreted easy to use with respect to the goal: no ‘strongly disagree’ score has been given by nobody, which is also the case of “Q1 = Overall, I am satisfied with how easy it is to use this system.” (M=4.76, Mdn=5, SD=1.25)

Similarly, a strong concentration of positive responses was observed for effectiveness and efficiency (Q3 = “I can effectively complete my work using this system” (M=4.69, Mdn=5, SD=1.95) – Q5 = “I am able to efficiently complete my work using this system.”: M=4.73, Mdn=5, SD=1.94), suggesting that participants generally felt able to complete their tasks successfully and without excessive effort. Learnability-related items (Q7 = “It was easy to learn to use this system”: M=4.9, Mdn=5, SD=1.69), Q8 = “I believe I became productive quickly using this system”: M=4.46, Mdn=5, SD=2.41) also showed robust agreement, indicating that users felt productive quickly and found the system easy to learn. This resulted in a strong overall satisfaction rating (Q19 = “Overall, I am satisfied with this system.”: M=4.55, Mdn=5, SD=1.8), with relatively few negative responses.

In contrast, a more mixed distribution emerged for items related to information quality and error handling (Q9 = “The system gives error messages that clearly tell me how to fix problems”: M=3.17, Mdn=3.5, SD=3.14 – Q14 = “The information is effective in helping me complete my work.”: M=4.18, Mdn=4, SD=3.6). While many participants still expressed agreement regarding clarity and usefulness of information (Q11 = “The information (such as on-line help, on-screen messages, and other documentation) provided with this system is clear.”: M=4.5, Mdn=5, SD=2.45), these questions exhibited a higher proportion of neutral and negative responses compared to other dimensions. In particular, feedback related to error messages and recovery (Q9, Q10 = “Whenever I make a mistake using the system, I recover easily and quickly.”: M=4.55, Mdn=5, SD=1.8) showed greater variability, suggesting that guidance provided during errors and recovery from mistakes may represent areas for improvement.

Q9 and Q10 received a low response rate because most users did not encounter any errors while using the system. As a result, they did not have the opportunity to assess whether the system corrected errors in a way that matched their expectations. Therefore, the limited number of responses to these questions should not be interpreted as a negative evaluation of the system’s error-handling capabilities. Q11 to Q15 focused on the quality of the information provided by the system. However, the user stories used during the evaluation were primarily task-oriented and focused on completing specific actions rather than reviewing or analyzing detailed information. As a result, users had limited exposure to information-rich content within the UI. The textual elements presented by the system were generally short, concise, and highly targeted to support task completion. Consequently, participants may not have had sufficient content to form a deeper impression of the quality, completeness, or usefulness of the information. Q16 to Q18 addressed such UI aspects and are conceptually similar to Q1 to Q3. However, the earlier questions were more explicitly focused on the system as a whole rather than on the UI itself. As a result, some participants may not have clearly understood the distinction between evaluating the overall system and evaluating the user interface. This potential ambiguity around the term “interface” may have contributed to lower response rates. In addition, participants may have perceived these questions as repetitive because they covered concepts already addressed in Q1–Q3, leading some respondents to skip them rather than provide similar answers twice.

In summary, the response distributions indicate a generally positive user experience, characterized by strong ease of use, learnability, and overall satisfaction, alongside more moderate evaluations of information presentation and error support. These results suggest that the system is largely usable and well accepted, while highlighting specific aspects of information quality that warrant further refinement.

### 7.3. Limitations and Threats to Validity

Several threats [99,100] have influenced the results of our gesture elicitation study, prototyping, and evaluation, which we discuss below.

#### 7.3.1. External Validity

Our GES was performed in a usability laboratory, not a real smart home, to ensure a varying level of control. Significant differences exist between them: the laboratory does not reproduce the real conditions, lacks the natural, long-term aspects and varying conditions that exist in a real home. Therefore, our empirical results should be interpreted with the understanding that they reflect a partial representation of the real world. Our IoT case study involved everyday object actions, also known as the “smart home”, to make it easier for participants to use the smart ring, instead of requiring them to learn a new environment. The representativeness of the results can affect their generalizability. The gestures analyzed in our GES can be considered a baseline. They cannot represent the whole design space of shared ring-based gestures. The 15 actions were selected to enable participants to complete the experimental session within a limited time, avoiding fatigue. Replications are envisioned with different setups.

Our study involved only N=30 participants to limit the sample size and to determine whether the ring-based gestures shared within a group were feasible. On the one hand, participants’ self-reported familiarity with devices such as computers or smartphones is comparable across the sample. On the other hand, variations in age and background introduce personal traits that have not been accounted for, which may introduce a confounding variable. A diversity of backgrounds among a limited set of participants can introduce a confounding variable if those background traits correlate with both the treatment and the outcome. We could have mitigated this risk by applying homogeneous sampling (e.g., participants with the same background) or stratified sampling (e.g., participants in the same age group). This would require a significantly larger sample size to avoid splitting a small sample into even smaller subgroups. As the population is characterized by a rich diversity of profiles, needs, and cultures, we should expand the experiment to replications concerning these aspects. For example, the smart ring could also be used by people with disabilities or of different ages (e.g., seniors might be interested in this technology according to Bilius et al. [8]). Similarly, while a sample of our size is sufficient to uncover major usability problems and good effect sizes, it cannot capture subtle population variances.

#### 7.3.2. Internal Validity

The learning effect, the understandability of the protocol, and the representativeness of Oringano must be considered. The learning effect was mitigated by randomizing the order of presentation of the referents. The understandability of the elicitation was assessed throughout the study. To avoid possible sources of bias, the materials were evaluated by an experienced researcher. We mitigated the instrumentation validity by using Oringano, a specific prototype. The GES procedure depends on two factors:

1. Participants were randomly exposed to one referent at a time and asked to consider one proposal only, independently. Some were easier to generate a gesture proposal for than others due to familiarity with the action and the legacy bias [101].
2. The experimenter has to correctly perceive and report each gesture proposal and verified that all 30 participants completed all tasks.

We did not consider the physical limitations of the smart ring (here, a Logbar Ring Zero), such as its form factor, size, color, or battery life. Although usability questions considered comfort, some participants did not answer the question because they were unsure whether they were evaluating the smart ring itself or the Oringano application, despite the project description. Several questions may have confused the participants and ultimately caused them to lose the evaluation. Several participants remained focused on the smart ring rather than the application.

#### 7.3.3. Construct Validity

The within-subject design adopted in the GES and the usability evaluation could be restructured as a between-subject design to mitigate potential learning effects that influence the process and the measurement of its results. The metrics and the participants’ apprehension about being evaluated must be considered. We mitigated the threat to metrics by using the CSUQ metrics, keeping them consistent. The participants’ possible apprehension about being evaluated was mitigated as they were informed that Oringano was assessed. However, the same participants have been involved in both the requirements engineering + GES (design) and the usability evaluation of the Oringano application supporting it (evaluation). This introduces an *ownership bias*: because participants evaluated an application they helped create, they were potentially more familiar with it, more forgiving of its defects, and prone to give better scores in the questionnaires. We mitigated this risk by insisting that the usability evaluation was on the management of shared ring-based gestures as supported by Oringano, instead of the gestures themselves. We could have applied a hybrid validation model in which the sample is divided into 70% participating only in the design part and the remaining 30% in the usability evaluation. The limited participant recruitment prevented us from doing so. As post hoc power calculations [102] generally provide limited additional information for interpreting the results of a completed study [103], we discussed the uncertainty associated with the estimates by reporting effect sizes and confidence intervals at 95%.

#### 7.3.4. Conclusion Validity

Data collection and the validity of the calculations must be considered. To mitigate threats to data collection, we systematically applied exact measurement and data extraction procedures across studies. The most appropriate calculations were performed by selecting them based on the experimental design and the nature of the variables and their hypotheses.

## 8. Design Implications

The GES (Section 5) and the Oringano prototype evaluation (Section 7) provide several implications for the design of shared, ring-based interaction techniques for smart-home environments. We suggest mainly five implications for designing these gestures.

### 8.1. DG1—Prefer Ring-Based Gestures with High Agreement for Frequently Used Actions

In principle, commands that are executed frequently by many users in the same group should be associated with gestures exhibiting the highest agreement rates. The GES showed that participants naturally converged toward similar gestures for concrete actions such as navigation, confirmation, media control, and object manipulation, resulting in an overall agreement rate of AR=0.272 (Figure 13 and Table 7), which is higher than the original study by Gheran et al. (AR=0.16) and previous replication studies (AR=0.09 and AR=0.228). These results suggest that high-agreement gestures are better candidates for standardization because they reduce learning effort and facilitate interoperability between users sharing the same smart-home ecosystem.

### 8.2. DG2—Exploit Natural Physical Metaphors

Participants predominantly proposed gestures based on familiar physical interactions with everyday devices. As illustrated in Figure 7 and substantiated in Section 5.3, particularly Table 5, rotational gestures were naturally associated with continuous parameter adjustment (e.g., volume or brightness), tapping gestures with confirmation, and directional movements with navigation or device selection. Furthermore, the distribution of gesture characteristics (Figure 8) reveals that dynamic gestures involving simple finger or hand movements largely dominated the elicited vocabulary. Designers should therefore exploit embodied interaction metaphors that preserve the semantic relationship between the performed movement and the intended command, thereby improving intuitiveness and memorability (Figure 12a).

### 8.3. DG3—Favor Gestures That Balance Recognition Accuracy (Performance) and User Acceptance (Preference)

Gesture design should jointly consider user preference and recognition accuracy: the ongoing debate regarding the balance between user preference and system performance applies here as well. The Oringano ring prototype demonstrated that simple rotational, translational, tapping, and pinching gestures generate distinctive inertial signatures that can be accurately recognized using embedded IMUs while remaining admissible to perform. Participants consistently reported high confidence in the gestures they proposed, with average Goodness of Fit close to the maximum of the five-point scale (Figure 12b). Moreover, memorability remained high across all referents (Minimum = 3.7/5, Maximum = 4.7/5) while perceived complexity remained relatively low (Figure 12a). These findings indicate that simple gestures simultaneously improve recognition accuracy and user acceptance.

### 8.4. DG4—Support Gesture Personalization for Abstract Actions

Although strong agreement emerged for concrete manipulation tasks, actions involving abstract semantics (e.g., enabling personal assistants or system-level functions) resulted in lower agreement rates and longer thinking times than direct manipulation tasks that are considered concrete actions. The GES results indicate that these actions are associated with multiple competing mental models rather than a single universally preferred gesture. Consequently, shared smart-home systems should adopt a hybrid vocabulary that is composed of (1) standardized gestures for high-frequency actions and (2) configurable or personalized gestures for actions that are suffering from lower agreement rates. Such adaptive action-gesture mappings can preserve consistency among group members without sacrificing individual preferences.

### 8.5. DG5—Reinforce Gesture Learning Through Immediate Feedback

The learnability and the discoverability of gestures are two critical aspects of gestural interaction [1,76]. The usability evaluation demonstrates the importance of immediate feedback when interacting through smart rings. The CSUQ results indicate positive evaluations across all usability dimensions, with every category scoring above 4.1/7 and usefulness obtaining the highest average (M=4.7, SD=1.43—see Figure 18). Participants also reported high overall satisfaction with the ring-based interaction, suggesting that visual confirmation provided by the prototype facilitated gesture execution and increased confidence. Future ring-based gestures should therefore integrate multimodal feedback, including visual, auditory, or haptic cues, to confirm successful recognition, reduce repeated gesture execution (e.g., when gestures are repeated and could be composed), and improve transparency in shared smart-home environments.

The sharing of ring-based gestures enables a common interaction language for smart-home environments. Our results show that participants converge toward similar gestures for many everyday actions, supporting the definition of a standardized core vocabulary that minimizes learning effort and promotes consistent interaction across users and devices. For actions exhibiting lower agreement, personalization should complement standardization to accommodate individual preferences and accessibility needs.

## 9. Conclusions and Future Work

To better understand how users associate ring-based shared gestures with smart home commands, this paper presented Oringano, a framework for designing and evaluating a vocabulary of shared ring-based gestures for controlling IoT devices in a smart home. These gestures are original in that members of the same group can share the same gestures for the same actions, as well as gestures customized by each individual.

First, we synthesized contemporary smart ring-based gesture interaction literature using a SLR to identify related GESs, to discuss the most influential GESs, and to compare four other reviews on ring-based gestures with respect to our study.

Second, we conducted a user-centered requirements elicitation to identify representative smart-home control actions for a smartphone application that would manage these actions by ring-based gestures. Thirty participants contributed to fifty-one user stories clustered into seven epics. Fifteen actions/referents have also been defined.

Third, we performed a gesture elicitation study to extend user-defined shared gesture inputs using a smart ring, offering an intuitive and usable interaction paradigm through a dedicated smartphone application, that was initiated in the previous stage. Unlike most systems that rely on predefined gesture sets, we support the creation of personalized gestures that can be shared among members of a group. This mechanism allows groups, such as families or communities with shared cultural practices, to establish a common gesture vocabulary tailored to their preferences. We analyzed the agreement rate, the thinking time, the goodness of fit, the complexity, and the memorability of these gestures proposed by the same thirty participants. The shared gestures proposed in this study benefit from a higher average agreement rate than other GESs with rings, but they also showed a slightly lower goodness of fit and a longer thinking time.

Fourth, we conducted a usability evaluation of Oringano, a prototype of the smartphone application responsible for managing shared gestures and using them to control IoT devices in a smart home. Our study shows that participants expressed interest in the smart ring’s potential in terms of the usability of the application using the elicited vocabulary. The major contribution of this Oringano framework is to offer some flexibility in sharing gestures created by users for a specific IoT action, thereby avoiding any additional cognitive load associated with learnability.

Although the study was limited to thirty participants with a good gender balance, future research could expand the sample size and make the study more inclusive to better assess its impact on the large representative population. More participants with conditions such as dyslexia or visual impairments would evaluate the suitability and potential of these shared gestures and the smartphone application. All these participants would evaluate and compare the use of the connected ring with these IoT devices in a real smart home, thereby posing questions such as investigating multi-user interaction, safeguarding critical actions, monitoring real usage over time, and performing a longitudinal study of the practice of sharing gestures: who are the users akin to administering the ring-based ecosystem, who are the users akin to customizing and sharing their own gestures, and how other users would accept or reject these additional gestures.

## Figures and Tables

**Table 1:** Comparison of the four SLRs on ring gestures.

**Subject**	Villarreal et al. [33]	Vatavu et al. [51]	Villarreal et al. [34]	Wang et al. [28]
**Year**	2020	2021	2024	2025
**No. cit.**	191	46	52	12
**SLR Method**	Prisma [54]	SLR inspired by [52] in a medicine library	Prisma [54]	SLR method with 2 sources in software, 1 in education, and Prisma
**Method precision**	“Radically different from conducting a survey or from free-form discussion of related literature”	Investigation of ring gestures and devices consists of three questions with qualitative method	Microscopic analysis gestures and referents from GESs compared to [33]	The SLR method is based on six steps designed to address the four research questions identified
**Corpus**	N= 216 papers	N= 84 papers for qualitative analysis of ring gestures	N= 267 papers	Out of 593 papers, N= 206 papers in analysis study
**Query**	“Gesture” AND “Elicitation” AND “Study”	Several queries depend on the stage of the SLR	“Gesture” AND (guess* OR elicit*) AND (study OR experience) in taxonomy research	Several queries depend on the stage of the SLR
**No. Rings**	Rings described in [4]	“ring devices,” “ring gestures”	Three types: rings for short, ring-like, handheld ready devices	Ring form-factor [58]
**Major Contribution (extension of SLR)**	Understand and compare how current and new GESs are positioned in the literature (into a web site available) and identify opportunities for unexplored areas	Web-based tool to collect/describe GES	A macroscopic analysis (terms and associations) + systematic categorization of referents, and gesture–referent mappings.	Medical tasks: The ring is associated with sensing—providing constant access to data such as heart rate, motion, and skin temperature—yet progress, for instance.

**Table 2:** The evolution of the most influential GESs, authors, and application domains related to smartphones, smart TVs, and recent works (up to 2025), based on their number of Google Scholar citations [34]. ✓ = a smart ring is used.

Authors	Title	Application	Smart Ring	Year	# Cit.	% Since 2024 from [34]
Ruiz et al. [74]	User-defined Motion Gestures for Mobile Interaction	Smartphones	✓	2011	624	+39.28%
Kane et al. [75]	Usable Gestures for Blind People: Understanding Preference and Performance	Tablets, smartphones	✓	2011	426	+32.29%
Kuhnel et al. [27]	I’m home: Defining and evaluating a gesture set for smart-home control	smart home		2011	278	+40.4%
Vatavu et al. [59]	User-defined Gestures for Free-hand TV Control	Smart TV		2012	291	+39.44%
Nacenta et al. [76]	Memorability of pre-designed and user-defined Gesture Sets	Tablets		2013	317	+ 61.73%
Zaiti et al. [73]	On free-hand TV control: experimental results on user-elicited gestures with Leap Motion	TV home		2015	121	not cited in table of [34]
Lou et al. [70]	Personalized gesture interaction for cyber-physical smart-home environments	Smart-home		2017	39	not cited in Table of [34]
Vogiatzidakis et al. [64]	Frame-based elicitation of mid-air gestures for a smart home device ecosystem	Smart-home		2019	30	not cited in Table of [34]
Stec et al. [71]	Gestures for controlling a moveable TV	TV home		2018	12	not cited in Table of [34]
Wu et al. [72]	Beyond remote control: Exploring natural gesture inputs for smart TV systems	TV home		2018	20	not cited in Table of [34]
Bilius et al. [50]	GestuRING: A Web-based Tool for Designing Gesture Input with Rings, Ring-Like, and Ring-Ready Devices	Web-based tool (compatible smartphone)	✓	2021	46	not cited in [34]
Vanderdonckt et al. [47]	Four Replications of a User-Defined Gesture Study with Smart Rings	Smart-home	✓	2025	1	recent

**Table 3:** Motivation and comparison of our GES to existing GESs.

	Original Study [4]	Replication [46]	Study on Older People + Smart Ring [8]	τ-ring [11]	Oringano (This Paper)
**Year**	2018	2023	2023	2025	2026
**Participants**	*N* = 24 (15 male and 9 female) between 21 and 45 years old (*M* = 27.5, *SD* = 7.9)	*N* = 24 (15 male and 9 female) between 22 and 45 years old (*M* = 26.3, *SD* = 5.7)	*N* = 9 older adults (5 male and 4 female) between 61 and 69 years old (*M* = 64.9, *SD* = 2.8)	*N* = 34 (for health rate monitoring) *N* = 3 (for handwriting text)	*N* = 30 (15 Male and 15 female, *M* = 37.53, *SD* = 15.31)
**Apparatus**	2 Ring Zero (https://web.archive.org/web/20170309191236/http://ringzero.logbar.jp/, accessed on 4 December 2017) devices, video recording	2 Ring Zero (https://web.archive.org/web/20170309191236/http://ringzero.logbar.jp/), video recording	3 commercial rings: one Ring Zero, one Litho (https://www.litho.cc/) and one Omni (https://omnihealthring.com/)	1 τ-ring	Multiple shared Ring Zero devices, smartphones, and IoT devices
**Design**	within-subjects with two independent variables: Referent and Num-Rings	within-subjects with two independent variables: Referent and Num-Rings			within-subjects with one independent variable, five independent variables
**Environment**	Home	Home			Smart home
**Referents**	*P* = 14, including “TV on/off,” “start player,” “volume up,” “lights on/off,” “go to the next item in a list”	*P* = 5: “TV on/off,” “start player,” “volume up,” “lights on/off,” “go to the next item in a list”	Ring used interacting with applications on any devices and streamlining access control and POS payments	Health sensors and handwriting tasks	*P* = 15 (Table 5)
**# Rings**	*one-ring* and *two-rings*, randomized across participants	*one-ring* and *two-rings*, randomized across participants	*one-ring in three different types* across between 61 and 69 years old participants	*one-ring* in commercial way	multiple, shared, rings, randomized across participants
**Smart Ring + other(s)**	Smart Ring + IoT	Smart Ring + IoT	Smart Ring + IoT + web-Application	Smart Ring + Application mobile	Smart Ring + IoT + Application mobile
**Gesture set**	672 elicited gestures, of which 240 for the five referents used in the replication study	240 elicited gestures corresponding to the five referents used for the replication	579 mappings between ring gestures and system functions for interactive applications, extracted from the 84 papers	N/A	450 gestures performed by *N* = 30 participants
**Measures**	AR [35], Thinking Time (seconds), Gesture-Goodness of Fit (10-point Likert scale)	AR [35], Thinking Time (seconds), Gesture-Goodness (10-point Likert scale rating)	Qualitative Research based on three questions	A recognition accuracy measure	AR [35], Thinking Time (seconds), Gesture-Goodness, quantitative usability tests, frequent use of digital domain, qualitative questions

**Table 4:** Three examples of user stories collected: a domain-oriented user story, a functional one, and a non-functional.

Property	Value
User story **US-04**	Epic 1
Priority	Should have
Title	Guidance
Description	As a user, I would like some guidance the first time I use the app so that I can better understand its technical aspects
Type	Domain
User story **US-06**	Epic 2
Priority	Must have
Title	Profile creation
Description	As a user, I would like to have my own profile so that I can access my personal information and preferences regarding settings, customize gestures and actions, and view my connected devices
Type	Functional
Dependency	US-05
User story **US-16**	Epic 3
Priority	Must have
Title	Database of common models of connected devices
Description	As a service provider, I need a database that lists all the necessary information about the common (most frequently used) connected devices in a user’s home, categorized by type (e.g., TV, lamp) and the actions supported by the app, so that the user can control their connected network using the ring
Type	Non-functional

**Table 5:** List of the 15 actions/referents defined before the study (first column) associated with the first and second most preferred ring-based gestures proposed by participants during the study (second and third columns). AR = Agreement Rate.

#	Referent	First Most Preferred Gesture (AR)	Second Most Preferred Gesture
1	Turn the TV on/off	Press a button (0.232)	Draw a letter or a figure
2	Turn the media player on/off	Bend the index (0.231)	Rotate the hand clockwise
3	Turn lights on/off	Half closed fingers (0.191)	Draw a letter or a figure
4	Turn personal assistant on/off	Draw a letter or a figure (0.207)	Press a button
5	Turn ring control system on/off	Draw a letter or a figure (0.256)	Press a button
6	Increase volume	Turn fingers clockwise (0.282)	Rotate the ring clockwise on the index
7	Decrease volume	Turn fingers counterclockwise (0.301)	Open hand – arm down
8	Play	Draw a letter “M” (0.294)	Swipe horizontally
9	Pause	Draw a letter or a figure (0.201)	Press a button
10	Stop	Bend a finger (0.164)	Swipe horizontally
11	Go to next item	Swipe horizontally (0.360)	Rotate the hand clockwise
12	Go to previous item	Swipe horizontally (0.352)	Rotate the hand counter-clockwise
13	Answer Yes/OK to system question	Draw a letter or a figure (0.439)	Half closed fingers
14	Answer No/Cancel to system question	Draw a check (0.350)	Swipe horizontally
15	Help/ask the system for assistance	Draw a question mark (0.552)	Rotate the index

**Table 6:** Categorization of some gestures proposed by participants: *range of motion* (i.e., Small, Medium, or Large), *nature* (i.e., Symbolic, Metaphorical, Physical, or Abstract), and *form* (i.e., Stroke, Static, Static with motion, or Dynamic).

Cat.	Gesture Name	Range of Motion	Nature	Form
1	Swipe horizontally	Medium	Symbolic	Stroke
2	Swipe finger up	Medium	Symbolic	Stroke
3	Swipe diagonally	Medium	Symbolic	Stroke
4	Swipe finger down	Medium	Symbolic	Stroke
5	Rotation	Medium	Symbolic	Stroke
6	Swipe hand open up	Medium	Symbolic	Stroke
7	Half closed fingers	Small	Physical	Static
8	Draw a letter or a figure	Large	Abstract	Dynamic
9	Index pointing downward	Small	Physical	Dynamic
10	Open hand-arm down	Large	Physical	Static with motion
11	Open hand-arm up	Large	Physical	Static with motion
12	Press a button	Medium	Metaphorical	Static with motion

**Table 7:** Comparison of our Oringano study against other GESs.

Criteria	Original GES [4]	Oringano GES	Difference of Percentage
Total number of elicited gestures	120	450	
Shared distinct gestures	n/a	12	
Average Agreement Rate	0.16	0.294	+84%
AR (TV on/off)	0.120	0.232	+93%
AR (start player)	0.076	0.232	+205%
AR (volume up)	0.130	0.282	+117%
AR (lights on/off)	0.040	0.191	+377.5%
AR (next)	0.225	0.360	+62.5%
Thinking Time	4.60	9.90	+115.2%
Goodness of Fit	7.91	6.91	−12.64%

**Table 8:** Group specifications: Attributes, Methods, and Class Relationships.

**Group Attributes**		
**Attribute Name**	**Type**	**Description**
creationDate	Date	Date of creation of the group.
groupName	String	Name of the group. This name is unique.
groupNickname	String	Nickname of the group, used to provide a more descriptive and human-readable name than the unique group name.
**Group Management Methods**		
**Method Name**	**Description**	
manageDevices	This method links the devices of a group with user profiles.	
manageAdmin	This method ensures that at least one administrator exists in the group. If the group has only one administrator and they attempt to leave the group, an error message is triggered.	
checkGroupName	This method verifies that the group name is unique. If it is already used by another group, an error message is triggered.	
**Class Relationships**		
**Class**	**Relationship Type**	**Description**
Device	Aggregation	Each group has 0..n devices.
Permission	Association	Permissions are centralized in the group as they are specific to a Profile–Device combination.
Profile	Aggregation	Each group has 0..n profiles. Deleting the group does not delete profiles.
AdminProfile	Composition	Each group has 1..n admin profiles. If the group is deleted, an admin profile becomes a regular profile again.

**Table 9:** The class personnalizedGestureSpecification: Methods and Class Relationships.

**Custom Gesture Methods**	
**Method Name**	**Description**
record2DGesture	Allows recording a new gesture and storing the corresponding 2D image in the image attribute.
setName	Allows the user to assign a name to their custom gesture.
**Class Relationships**	
**Class**	**Description**
Profile	Aggregation: each custom gesture is associated with a profile.
Action	Composition: a custom gesture may be linked to an action. If the action is deleted, the custom gesture is also deleted.
DefaultGesture	Inheritance: the custom gesture inherits from the DefaultGesture class.
Activity	Composition: a custom gesture may be linked to an activity. If the activity is deleted, the custom gesture is also deleted.

## Data Availability

The original contributions presented in this study are included in the article. Further inquiries can be directed to the corresponding author.

## Abbreviations

The following abbreviations are used in this manuscript:

AR	Agreement Rate
BLE	Bluetooth Low Energy
CSUQ	Computer System Usability Questionnaire
GDPR	General Data Protection Regulation
GES	Gesture Elicitation Study
HCI	Human–Computer Interaction
HMM	Hidden Markov Model
IBM	International Business Machines
IMU	Inertial Measurement Unit
IoT	Internet of Things
NFC	Near-Field Communication
Oringano	**O**pen **RIN**g-based **G**esture interaction in **AN** internet **O**f Things
PICO	Population, Intervention, Comparison, and Outcome
PRISMA	Preferred Reporting Items for Systematic Reviews and Meta-Analyses
SLR	Systematic Literature Review
RQ	Research Question
SVM	Support Vector Machine
US	User Story
VR	Virtual Reality

## Appendix C. User Scenario Conducted During the Experiment

**Scenario and Tasks**	**Description**
Scenario	We’ll follow the adventures of a family to help you discover our Oringano application in a fun way. This application enables you to control smart home devices by performing ring-based gestures that can be managed through the application and shared among family members. Let’s meet our family below. The mom’s name is Marie. She’s 42 and a doctor. She’s married to John, a 45-year-old car salesman. They have a 14-year-old daughter and a lively 12-year-old son. Their names are Caroline and Peter, respectively. After hearing about the connected ring from a family friend, they were all drawn to the convenience the ring could offer and the new user experience it promises. They live in a house where all their connected devices are gathered in the living room, and they want to share this experience—but only with people they’ve personally approved. In fact, they want to share the ring with their children whenever possible. They also believe that the ring should be shared with family and not be taken outside their home. The ring is the gadget that makes it easier to use the smart devices in their living room, and they need to manage access.
Creating Marie’s profile	After purchasing the ring and downloading the app, Marie must enter the license number to log in for the first time. Once the license number is entered, she can create her profile with her settings.
Creation of Marie’s group	Marie chooses a nickname for the group and adds her family members using a list that enables people whose email addresses are entered to find the group when they go to the “Join Group” tab. Creating the group automatically displays the screen for setting up devices. This lets her know she’s in the right place. In this step, she must use the device detection feature, which works via Wi-Fi. This feature allows Marie to access a list of all devices connected to her network. She selects the detected device she wants to add to the group and is then taken to a screen listing all the apps for the connected devices. To configure a device, Marie is asked to log in to the app designed for her connected device. This is what allows the ring’s app to communicate with the devices. After configuring her devices, Marie will finally be able to record gestures for the various actions each device can perform. To do this, she will need to sync her ring. We’ll explain this step when we cover the app’s daily use. Once the ring is synced, she’ll be able to access the main menu using the “Home” button. All she’ll need to do is go to the “Action” category.
Configuring an action and recording a gesture	Marie selects the device she’s interested in from a list. This allows her to view all the actions that device can perform directly in a second list. From there, Marie goes to the options in the “Actions” category. She adds up to two custom gestures or sticks with the default gesture that is suggested to her
Configuring an activity as a sequence of actions	Marie is browsing the app and discovers the “Activity” tab. As explained in the manual, an activity allows you to trigger a sequence of actions. Every night, she watches a movie with her family, and to do so, she has to perform three actions: “Turn off the lights”, “Turn on the TV”, “Turn on the media player”. She thinks it would be faster to perform all of them with a single gesture. Thanks to the “Activity” option, Marie can do just that. All she has to do is select the actions she wants for the connected devices she needs. Then, she must record a gesture that will be associated with this sequence of actions. Marie records them as an activity under the name “watching TV.” Finally, she associates the action with it in the same way she would for any other action.
Creating Marie’s guest profiles—Join Marie’s group	Everyone arrives, and Marie shows the others how to use the app. They download it too, and each of them creates a profile. Thanks to the email addresses Marie added when she created the group, the group appears on the last screen below when family members click “Join Group.”
